# Factors Affecting Multiple Paternity: Insights From the Eastern Quoll (*Dasyurus viverrinus*)

**DOI:** 10.1002/ece3.73515

**Published:** 2026-04-30

**Authors:** Brittany M. Brockett, Linda E. Neaves, Maldwyn J. Evans, Iain J. Gordon, Jennifer C. Pierson, Belinda A. Wilson, Claire Wimpenny, Adrian D. Manning

**Affiliations:** ^1^ Fenner School of Environment and Society The Australian National University Canberra Australian Capital Territory Australia; ^2^ School of Environmental and Conservation Sciences Murdoch University Perth Western Australia Australia; ^3^ James Hutton Institute Dundee UK; ^4^ Central Queensland University Townsville Queensland Australia; ^5^ College of Science & Engineering James Cook University Townsville Queensland Australia; ^6^ Australian Wildlife Conservancy Subiaco East Western Australia Australia; ^7^ Centre for Conservation Ecology and Genomics, Institute for Applied Ecology University of Canberra Canberra Australian Capital Territory Australia; ^8^ Office of Nature Conservation ACT Government Dickson Australian Capital Territory Australia

## Abstract

Breeding systems influence population dynamics and genetic diversity, especially when a population is small or isolated. To examine population‐level drivers of multiple paternity, we combined long‐term demographic data and 1745 single‐nucleotide polymorphisms from a reintroduced population of eastern quolls ( *Dasyurus viverrinus* ). We also conducted a cross‐species comparison of multiple paternity occurrences within the Dasyuridae (including the eastern quoll) to look for taxonomic patterns. Pedigree reconstruction revealed high rates of multiple paternity, with 47%–85% of litters sired by more than one male (depending, respectively, on whether all litters or only those with > 1 offspring were assessed). Reconstructed litters contained up to three sires. Trait‐based generalised linear mixed modelling showed individual reproductive success was significantly correlated with higher bodyweight, lower age, and having a fawn, rather than black, colour morph. Across the Dasyuridae family, higher rates of multiple paternity were associated with shorter lifespans, but not sexual size dimorphism or greater intersex aggression. Our findings indicate that individual traits predict reproductive success in the eastern quoll, and that within a polyandrous taxonomic family, the likelihood of multiple paternity can depend on the evolutionary life history of the species (e.g., lifespan).

## Graphical abstract

Eastern quolls ( *Dasyurus viverrinus* ) exhibit multiple paternity in approximately half of all litters. Reproductive success is correlated with heavier weight, youth, and being black rather than fawn colour morph. A cross‐species analysis indicates that high rates of multiple paternity within the Dasyuridae area correlated with shorter lifespan and increased rates of polyandry, but not sexual size dimorphism.

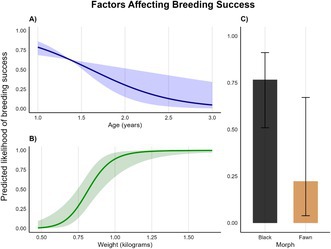

## 1 Introduction

Breeding systems in mammals are varied, ranging from monogamy to promiscuity, from generating one offspring at a time to mass production, with diverse sub‐classifications and combinations shaped in part by life history, behaviour, and ecological context (Emlen and Oring 1977; Clutton‐Brock 1989, 2021). Understanding how these systems operate is particularly important in small, isolated populations, where mating structure can strongly affect genetic composition and demographic stability.

Multiple paternity is relatively well studied—it is a promiscuous, polyandrous mating system in which a female will mate with, and wherein a single litter is sired by, multiple males (Sugg and Chesser 1994; Taylor et al. 2014). Polyandry can reduce relatedness among offspring (Glen et al. 2009), increase offspring survival and female lifetime reproductive fitness, and limit reproductive monopolisation by a small number of males (Fisher, Double, et al. 2006). Population‐level effects are likely to be most pronounced in small populations (Pearse and Anderson 2009), although extremely low population densities and encounter rates may constrain multiple male mating where it may otherwise occur at higher rates (Sale et al. 2013). Proposed evolutionary drivers of polyandry include the avoidance of intersexual aggression or infanticide (Wolff and Macdonald 2004), increased offspring quality, greater genetic diversity, and female bet‐hedging against mate quality (Jennions and Petrie 2000).

Multiple paternity adds further complexity to polyandry, with diverse life history and population interactions. Though polyandry often occurs where there is sexual size dimorphism (Weckerly 1998), multiple paternity is likely to be least frequent in populations where sexual dimorphism is extreme (Dobson et al. 2024). A review by Correia et al. (2021) presented evidence for multiple paternity both increasing and decreasing the strength of sexual selection, increasing post‐copulatory sperm competition, and affecting social cooperation. Meta‐analyses across 59 mammalian species indicated weak correlation between litter size and both the probability of multiple paternity and the number of sires per litter (Dobson et al. 2018), but this relationship is likely moderated by behaviours that restrict female mate choice, such as mate guarding and territoriality (Correia et al. 2021). Generally, there is a positive association between litter size and the probability of multiple paternity occurring (Correia et al. 2021). However, where mating success is highly uneven, observed rates of multiple paternity may be lower than expected based on litter size alone (Correia et al. 2021). It is therefore important to view multiple paternity within the context of the species‐specific traits that may affect breeding systems, to fully understand their effects on population dynamics and evolution.

The marsupial family Dasyuridae provides a valuable system for examining these interactions since it is relatively well studied, polyandry is widespread, and many members exhibit semelparity; a life history characterised by a post‐breeding die‐off of adult males. Semelparity is thought to arise from intense sexual competition, with males maximising reproductive effort at the expense of somatic maintenance (Naylor et al. 2008; Fisher et al. 2013; Gaschk et al. 2023). In systems such as these, where male reproductive success is highly competitive, their breeding lifespans are often shortened as compared with females, and males are rendered more susceptible to physiological and environmental stressors (Clutton‐Brock 2021). In extreme cases, such as the *Antechinus* genus, the elevated testosterone and stress of the breeding season lead to multiple organ system failure (Naylor et al. 2008). Under these conditions, theory predicts that strong sexual selection should skew reproductive success towards a small number of males, resulting in lower‐than‐expected rates of multiple paternity (Correia et al. 2021).

Here, we examined the breeding system of the eastern quoll ( *Dasyurus viverrinus* ), a dasyurid reintroduced to a conservation‐fenced reserve, and identified individual‐level predictors of reproductive success. We then placed our findings in a broader evolutionary context by comparing patterns of multiple paternity across the Dasyuridae family in relation to life history, behavioural, and physiological traits and reveal new potential predictors of multiple paternity.

## 2 Materials and Methods

### 2.1 Dataset Generation

We investigated the breeding system of a reintroduced population of eastern quolls at Mulligans Flat Woodland Sanctuary (MFWS; Australian Capital Territory, Australia) to determine whether morphological or genetic factors affect individual reproductive success and rates of multiple paternity in the population. Reintroduction monitoring procedures were adapted from the protocols developed for eastern bettong ( *Bettongia gaimardi* ; Manning et al. 2019; Wilson 2023). Eight capture‐mark‐recapture trapping sessions were conducted annually, with 92 wire cage traps spaced approximately 200 m apart across MFWS to achieve broad spatial coverage. Traps operated for two consecutive nights, and each trapping session included a night of free feeding to minimise trap shyness and improve recapture probability (Wilson et al. 2023). Trapping sessions occurred in summer (to detect post‐juvenile dispersal) and autumn (to monitor the size of the breeding population) each year, with exact timing varying with weather conditions, In addition to general monitoring sessions, there was also a targeted den‐site trapping programme as part of post‐translocation monitoring where dams and offspring were captured together (2016–2018; Wilson et al. 2020).

Morphometric data collected included body weight, foot length (*PES*), head length, a pouch description (including presence/number of pouch young), an age estimate, and a general condition score. For downstream analyses, if an individual was captured multiple times within a year, then an average of the morphometric data for the year was used.

### 2.2 Genotyping and Filtering

To generate genome‐wide markers suitable for pedigree reconstruction, Diversity Arrays Technology (DArT) extracted and genotyped samples using Dasyurid DArTseq 1.0, an optimised complexity reduction technique modified from Kilian et al. (2012). We prepared and submitted samples following DArT's specifications, and the resulting sequence data were processed using DArT's proprietary analytical pipelines to identify candidate single‐nucleotide polymorphisms (SNPs). These candidate SNPs were then provided to us for project‐specific filtering. Few individuals from 2020 are represented in the dataset due to logistic limitations.

We filtered the 7142 candidate SNPs for quality and informativeness in R v 4.1.0 (R‐Core‐Team 2021) using the *dartR* package (Grueber et al. 2018; Mijangos et al. 2022). First, locus call‐rate and minimum acceptable repeatability thresholds of 0.95 were applied, ensuring that loci were represented in 95% of samples and that SNPs were not the result of sequencing errors. A minor allele frequency threshold of 0.05 was set, with various thresholds tested for their downstream population structure effects (Brockett et al. 2022). We applied aa minimum sequence depth of 10, and an allele depth ratio maximum of two was implemented to remove SNPs where one allele was sequenced at much greater depths than the other. The inbuilt dartR function gl.filter.secondaries was used with the method set to ‘best’, to retain only the SNPs with the highest polymorphic information content where multiple SNPs were present in a single tag. Following this locus‐based filtering, 1746 candidate SNPs and 171 individuals remained. We then removed individuals with call rates < 0.90 (*n* = 7). Finally, we removed any monomorphic loci generated by other filtering processes (*n* = 1). We did not filter SNPs based on Hardy–Weinberg Equilibrium because the dataset comprised samples from multiple, non‐admixed populations with large amounts of immigration—breaking many of the equation's assumptions. The final dataset contained 1745 SNPs across 164 individuals.

A single individual (88C7E) was removed from the dataset after SNP filtering due to potentially spurious breeding data (being a single male that was recorded as being 200 g in weight when first calculated to have reproduced, which is highly unlikely and attributed to a scribing error).

### 2.3 Pedigree Construction

Both demographic and genetic data were used to construct a pedigree of 164 eastern quolls in Colony v 2.0.6.6, a programme which uses maximum likelihood methods to simultaneously infer parental and sibling relationships (Jones and Wang 2010). The mating system was set to allow polygamy for both males and females, and because the population was not thought to have high levels of inbreeding, the non‐inbreeding model was used. We used a single long run and full‐likelihood methods to construct the pedigree. Allele frequencies were inferred by the programme and were not updated throughout the pedigree's construction based on inferred relationships, both to save computational time and because families were not thought to be extremely large. A weak sibship‐prior was set, with maternal and paternal priors set to 1 as the number of offspring likely to be produced by each sex was unknown. Since full sibships were thought to consist of a small number of individuals (< 20), sibship scaling was set to ‘no’. A single run was used, and the random seed was set to 1234.

The 1745 SNPs remaining after dataset filtration were provided as co‐dominant genetic markers, with a specified error rate of 0.02. We used demographic data to assign sex and constrain parentage to biologically plausible candidates. To do this, we first excluded all individuals in the dataset as potential parents for the Tasmanian founders. Next, we inferred birth years from estimated age‐at‐capture, excluded candidate parents that had not reached sexual maturity (≥ 10 months old) in the breeding season preceding an individual's birth, or were unlikely to be alive at conception (> 4 years for males, and > 5 years for females). Where field observations confirmed maternal relationships and sibships, we assigned these to improve pedigree accuracy. No paternal sibships were known a priori.

### 2.4 Investigation of Reproductive Parameters

We defined reproductive success as recruitment of offspring into the monitored population, rather than conception, birth, or pouch occupancy. Our genetic dataset necessarily excluded young that died before independence and individuals that evaded capture, and so we expected to have missed some offspring born at MFWS. However, moderate–high adult recapture rates (56.4% for females, 70.37% for males; Wilson 2023) indicated high sampling coverage. By focusing on recruitment, we quantified realised genetic contribution to the population. Where relevant, we discuss how incomplete detection may influence interpretation of multiple paternity and reproductive skew.

Female eastern quolls produce only one litter per year (Godsell 1983), with approximately 20 altricial young born but only six able to attach to the available teats (Hill and O'Donoghue 1913). This allowed us to manually group MFWS‐born individuals into generational cohorts based on birth year and pedigree‐assigned dam and sire. Using the reconstructed pedigree, we quantified the number of sires per litter, the proportion of litters exhibiting multiple paternity, the mean number of sires per litter, and the proportional contribution of each sire where there were multiple sires. To assess the potential effect of sampling limitation, we also recalculated multiple paternity rates for subsets of litters exceeding increasing size thresholds (e.g., > 2 young, > 3 young). Other reproductive parameters of interest in this study included at which age, over how many years, and with how many partners offspring were produced.

### 2.5 Predictors of Reproductive Success

We analysed predictors of reproductive success using generalised linear mixed modelling in the R package *glmmTMB* (v1.1.13; Brooks et al. 2017). We modelled successful recruitment (success/failure to breed) against the fixed‐effects sex, colour morph, individual heterozygosity, interactive effects of weight and age, including a quadratic weight term to accommodate non‐linear relationships. We included weight and age as interactive effects because data indicated that eastern quolls generally grow heavier with age, but older individuals lose weight as they become less fit. We included random effects of individual identity and year to account for repeated measures and year‐to‐year variation, respectively, and standardised continuous predictors (age, body weight, heterozygosity) to enable effect‐size comparisons.

The model was tested for homogeneity, overdispersion, collinearity, and overall model fit using the *performance* package (v0.15.3; Lüdecke et al. 2021). Using the dredge function of the *MuMIn* package (v1.48.11, Barton 2023), we evaluated all supported combinations of fixed effects, facilitating selection of the ‘best’ model. Note that because the model failed if NAs were present, each candidate model used the same dataset and was directly comparable. We used Akaike's Information Criterion (AICc; Burnham and Anderson 2002) model selection, retaining the most parsimonious model and preferring fewer parameters where ΔAICc was < 2, while models with ΔAICc < 5 were retained for comparison (Tables S1–S3). We verified convergence of the selected model using the performance package (Lüdecke et al. 2021). To visualise predicted relationships, we used *ggplot2* package (v4.0.1; Wickham 2011).

Due to model convergence preventing the inclusion of sex as an interactive term with body weight and age, we also fitted sex‐specific models using the same workflow to assess whether predictors differed between females and males.

### 2.6 Predictors of Individual Fecundity

To examine predictors of offspring number among breeding individuals, we fitted zero‐inflated Poisson models using a pseudo‐hurdle model approach to separate breeding occurrence from variation in offspring number among breeders. Our model included the fixed‐effects age, morph, sex, and cluster, with random effects of year and individual identity. We did not include weight as a predictor due to limited sample data. The model selection method described for the binomial success/failure model was implemented, and we again visualised the effects of significant variables on offspring number using *ggplot2*. This approach allowed us to use a larger sample size while still examining key biological predictors of reproductive output.

### 2.7 Cross‐Species Breeding System Comparison

To investigate factors that might affect the occurrence and pattern of multiple paternity, we collated data for 11 species within the Dasyuridae family ( *Antechinus agilis* , *A. minimus* , *A. stewartii* , *Dasykaluta rosamondae* , *Dasyurus geoffroyii, D. hallucatus*, *D. maculatus* , *D. viverrinus*, *Phascogale calura* , *P. tapoatafa, Sarcophilis harrisii*) and two outgroups of non‐dasyurid Australian marsupials for which multiple paternity information was available (*Acrobates pygmaeus, Tarsipes rostratus*). We searched using combinations of the terms ‘polyandry’, ‘semelparity’, ‘life history’, ‘breeding systems’, ‘multiple paternity’, ‘Dasyuridae’, and ‘dasyurids’. We then extended coverage via a partial snowball search method, wherein references within selected papers were in‐turn selected for investigation. We used the online tool Litmaps to check the resulting citation network for gaps, and we incorporated relevant suggestions into our corpus (Litmaps 2024). The results of this literature search are summarised in Figure S1.

Of the traits discussed in the literature we selected antagonistic breeding behaviours and sexual size dimorphism, both traits that are generally expected to predict polyandrous breeding systems (Wolff and Macdonald 2004), as likely predictors of multiple paternity frequency. We qualitatively categorised intersex aggression into a three‐point scale (1 for low aggression like some chasing behaviours, 2 for moderate aggression such as occasional biting or observed forced copulation, and 3 for high aggression such as frequent and severe biting or documented infanticide). If it was unclear which category was appropriate based on the available descriptions, we averaged the score (e.g., low–moderate scored 1.5). We quantified sexual size dimorphism as a ratio of male to female body weight difference, to enable a standardised comparison between species. We included litter size in our analysis because larger litters may provide greater opportunity for multiple paternity to occur and be detected. We also incorporated lifespan which, while not commonly considered in polyandry studies, we theorised may constrain dasyurid breeding systems given the prevalence of semelparity.

We used linear regression to investigate the relationships between each trait and the frequency of multiple paternity reported in the literature. For the eastern quoll, we used the frequency of multiple paternity in litters containing more than two young in these analyses, to ensure comparability with estimates from other species.

## 3 Results and Discussion

### 3.1 The Breeding System of the Eastern Quoll

#### 3.1.1 Reproductive Parameters

Our reconstructed pedigree (Table S4) revealed that 42 eastern quoll litters were successfully recruited into the MFWS population between 2016 and 2020. The mean number of young recruited by a dam was 2.54 (ranging 1–6) and most litters (83.3%) contained between one and three young (Table 1). The eastern quoll pedigree revealed that 47.62% of these successful eastern quoll litters in MFWS were sired by multiple males (Figure S1). Of the litters that exhibited multiple paternity, 75% had two sires, 20% had three sires, and 5% had four sires (Table 1).

**TABLE 1:** Characteristics of the eastern quoll ( *Dasyurus viverrinus* ) breeding system within Mulligans Flat Woodland Sanctuary (MFWS), ACT, Australia.

Parent characteristics	Male				Female			
	Min	Max	Med	Mean	Min	Max	Med	Mean
Reproductive age	1	3	1	1.18	1	2	1	1.20
Litters produced	1	10	1.5	2.46	1	2	1	1.07
Age of successful parent	1	3	1	1.43				
Number of mates	1	10	1.5	2.46	1	5	1	1.74
Number of offspring	1	17	2	3.57	1	6	2	2.54

Litter characteristics	Count	% all litters	Mean number of sires	% each litter size with multiple paternity
Litter size of 1	10	23.8%	1	Not applicable
Litter size of 2	16	38.1%	1.38	37.5%
Litter size of 3	9	21.4%	2.00	88.89%
Litter size of 4	5	11.9%	2.40	80.00%
Litter size of 5	1	2.4%	3.00	100%
Litter size of 6	1	2.4%	3.00	100%

Summary of breeding system data			
Unique mothers	39	Litters with multiple paternity	47.62%
Unique fathers	28	Litters > 1 young with multiple paternity	62.50%
Offspring	100	Yearly rate of litters with multiple paternity	41.20%
Mean Litter Size	2.38 (1–6)	Yearly rate of litters with > 1 young with multiple paternity	54.91%

Paternity evenness	1 sire	2 sire	3 sire	4 sire
Count	22	15	4	1
Mean Litter size	1.68	2.73	4.50	4.00
% Male 1	100.0%	61.1%	58.4%	25.0%
% Male 2	—	38.9%	36.9%	25.0%
% Male 3	—	—	23.8%	25.0%
% Male 4	—	—	—	25.0%

*Note:* Data derived from a pedigree constructed in Colony v 2.0.6.6. Pedigree construction was informed by 1745 single‐nucleotide polymorphisms and demographic data obtained through long‐term monitoring of the MFWS population.

Multiple paternity was detected more frequently when there were more offspring in a reconstructed litter (Table S5). When all 42 litters were considered, 47.62% exhibited multiple paternity. However, 62.5% had multiple paternity when single‐offspring litters were excluded (32 litters remaining). This pattern was consistent; 87.5% litters with > 2 offspring, 85.71% litters with > 3 offspring, and 100% litters with > 4 offspring had multiple paternity (correlation coefficient = 0.73, *p* < 0.005). This pattern means that sampling limitations (both due to unsampled individuals and failure of offspring to recruit) could artificially lower the calculated frequency of multiple paternity in the population. Nearly, half of all reconstructed litters contained a single recruited offspring, and therefore multiple paternity could not have been detected in our study. So, if the dams of these litters recruited more than the one offspring we captured in our dataset, and because capture data shows that pouch occupancy was on average higher than the recruited litter size we detected (4.95 compared with 2.54 offspring, respectively), it is likely that our study presents an underestimate. However, there is value in reporting the observed rate of multiple paternity in the recruited population, while also accounting for under detection and acknowledging sampling limitations. Therefore, we consider that eastern quolls have a multiple paternity frequency of between 47.62% and 87.50% (Table S5), but that further studies are needed to tease out the intricacies of multiple paternity frequency between birth, pouch deposition, and post‐dispersal recruitment.

We observed 39 dams and 28 sires (Tables S1 and S2). Nine known‐age dams successfully reared a litter after their first year (eight at 2 years, one at 3 years), though only two females had more than one litter. Therefore, most females raising young after their first year likely did not successfully reproduce in their first year. The average age of a reproductively successful female eastern quoll was 1.20 years (median = 1 year). Males also successfully recruited most offspring within their first adult year (mean 1.18 years, median = 1 year). Nine known‐age sires recruited litters at 2 years old, and a single male recruited a litter at 3 years old. Eastern quolls in Tasmania also exhibit the greatest reproductive success in their first 2 years (Godsell 1983), and a recent study by Morrison et al. (2025) found that three of seven sampled litters in Tasmania had either two sires (two litters) or four sires (one litter). These results are encouraging because they provide evidence that the eastern quoll breeding system within the 485‐ha conservation‐fenced MFWS is like that of remnant, wild populations in Tasmania.

The reconstructed pedigree produced placeholder parents (6/39 dams, 11/28 sires) and earlier studies have demonstrated that capture probabilities vary among individuals (Wilson 2023). Due to these (and previously discussed) sampling limitations, it is possible that the reproductive parameters that we report in Table 2, and discuss above, underestimate reproductive lifespan and patterns of paternity. This would mean that eastern quolls could feasibly recruit offspring for longer than we report, and with a greater variety of partners. It is likely that any underreporting is minimal, though, given alignment with previous studies on quoll reproduction (e.g., Godsell 1983; Morrison et al. 2025) and that our data represent a robust, long‐term, and relatively high‐density sampling regime. We believe that this study provides a reasonably accurate indication of reproductive patterns within the MFWS eastern quoll population, though as discussed for multiple paternity frequency, further study should address the limitations we report here.

**TABLE 2:** Effects of generalised linear mixed model predictor variables on reproductive success and fecundity in eastern quolls ( *Dasyurus viverrinus* ) at Mulligans Flat Woodland Sanctuary, ACT, Australia.

Predictors	Reproductive success (all individuals)			Male reproductive success			Female reproductive success			Individual fecundity (Poisson model)		
	Odds ratio (95% CI)	*Z*‐statistic	*p*	Odds ratio (95% CI)	*Z*‐statistic	*p*	Odds ratio (95% CI)	*Z*‐statistic	*p*	Incidence ratio (95% CI)	*Z*‐statistic	*p*
Intercept	**3.28 (1.04–10.34)**	**2.03**	**0.043**	**7.04 (1.03–48.16)**	**1.99**	**0.047**	0.41 (0.12–1.37)	1.44	0.150	**2.74 (1.62–4.30)**	**3.91**	**< 0.001**
Age (years)	**0.30 (0.14–0.67)**	**−2.93**	**0.003**	0.21 (0.03–1.73)	−1.44	0.149	0.38 (0.13–1.10)	1.78	0.075	n/a		
Morph (fawn)	**0.09 (0.02–0.43)**	**−3**	**0.003**	0.06 (0.00–1.94)	−1.58	0.114	0.21 (0.34–1.45)	1.65	0.099			
Heterozygosity	n/a			n/a			0.63 (0.28–1.42)	1.12	0.265			
Weight (kg)	**12.24 (3.89–39.31)**	**4.21**	**< 0.001**	7.15 (0.98–52.14)	1.94	0.052	**4.25 (1.54–11.71)**	**2.80**	**0.005**			
Weight (kg) quadratic	**0.5 (0.28–0.88)**	**−2.39**	**0.017**	n/a			n/a					
σ^2^	3.29			3.29			n/a (zero‐inflated Poisson model)			0.28		
ID	0.00			0.07			0*			0.15		
Year	0.00			0.00			0*			0.15		
Marginal *R* ^2^/Conditional *R* ^2^	0.602 / n/a			0.580 / N/A			n/a			0.000/0.515		
Number of unique individuals	52			19			33			24 (12 males, 12 females)		
Number observations	73 (across 4 years)			29 (across 4 years)			44 (across 4 years)			31 (across 4 years)		

*Note:* Reproductive success models (binomial) examine whether individuals successfully recruited offspring into the population. Reproductive success models are based, respectively, on all individuals for which appropriate data were collected (column 1), only males with appropriate data (column 2), or females with appropriate data (column 3). The individual fecundity (Poisson model, column 4) examines the number of offspring recruited among breeding individuals. Odds ratios (OR) indicate the relative likelihood of reproductive success; incidence ratios (IR) indicate the relative change in offspring number. Significant effects (*p* < 0.05) are shown in bold.

#### 3.1.2 Semelparity and Paternity Evenness

Our results showed that eastern quolls possess partial semelparity, as observed in other dasyurids. From 2018 onwards, approximately, 33.2% of the population was aged two, and in 2019–2020, approximately 14.15% were 3 years old. We observed two females having more than one litter, eight males that bred in 2 years, and two males that recruited young across 3 years. However, given that some individuals breed for more than one season, the eastern quoll does not possess true obligate semelparity as seen in many *Antechinus* species (wherein all adult males, and many females, die following a single breeding season), but rather exhibits partial semelparity as seen in the kaluta ( *Dasykaluta rosamondae* ; Hayes et al. 2019) and northern quoll ( *Dasyurus hallucatus* ; Oakwood 1997). The survival rate for eastern quolls is high in comparison to other dasyurids. Where approximately 5% of kalutas and 10% of northern quolls survive for more than 1 year, with unknown reproductive success, our long‐term monitoring data indicate that approximately 47% of the adult population of eastern quolls at MFWS may be over 1 year old each year (Table S4; Manning, *unpublished data*). There is some evidence that semelparity does not always occur at a fixed‐rate; in a fragmented system with few breeding opportunities and abundant resources, up to 50% of male northern quolls were found to survive to a second year (Cowan et al. 2024). The MFWS eastern quoll population established in 2016 under good conditions (high resource availability, no mammalian predators; Wilson et al. 2020; Wilson et al. 2021), but the high survival rate was consistent through time and persisted through the 2019–2020 drought conditions (Wilson et al. 2023). We therefore do not expect that the rates of semelparity would increase significantly in the MFWS population under changed environmental conditions.

Within the MFWS population there was a tendency for uneven siring success; a single male sired approximately 60% of young in a litter (Table S4; Figure S2). Siring unevenness has not been extensively studied in dasyurids but is well known in at least the agile *Antechinus* ( *Antechinus agilis* ), where a single male can sire up to 60% of the young in a litter (Fisher, Double, and Moore 2006). While detailed observations of male–female interactions were not possible in our study, Fisher, Double, and Moore (2006) determined that for the agile *Antechinus* it was always the last male mated with that would sire most of a females' litter. This ‘last‐male dominance’ is evidence of behavioural mechanisms males exhibit to maximise their breeding success within a system driven by sperm competition. Other behavioural mechanisms to overcome the effects of sperm competition include long copulatory periods (agile *Antechinus*; Shimmin et al. 2000) and repeated matings (swamp antechinus *A. minimus* ; Sale et al. 2013). Given that female eastern quolls can store sperm for up to 14 days (Taggart et al. 2003), equal to storage times in the agile *Antechinus* (Shimmin et al. 2000), it seems likely that at least some of these behavioural mechanisms would also be present in the eastern quoll. Additionally, if females are not prevented from mating due to intersexual aggression, their competitive breeding system would be expected to result in higher rates of multiple paternity (Correia et al. 2021).

The promiscuity observed in dasyurids likely benefits both sexes. While polyandry may allow females to hedge against mate quality (Jennions and Petrie 2000) or to avoid harassment and infanticide (Wolff and Macdonald 2004), it also increases the probability that males recruit any offspring at all. Kraaijeveld et al. (2003) demonstrated that, when there were approximately 20% offspring survival and 70% female survival, a male agile *Antechinus* would need to mate with at least six females to have a greater than 80% chance of recruiting offspring to the next generation. Eastern quoll adult survival is high but there is a 30% attrition rate between pouch attachment and recruitment to the adult population (Godsell 1983). The eastern quoll therefore has a highly competitive breeding system, which we expect to favour promiscuity (females mating with many males, sometimes multiple times).

#### 3.1.3 Reproduction Across Time

Pedigree reconstruction demonstrated that females tended to only produce one litter and recruit an average of 2.54 young in their lifetime (median 2, range 1–6), usually from a single litter, while males recruited an average of 3.57 offspring (median 2, range 1–17) across an average of 2.46 litters (median 1.5, range 1–10; Table 2, Table S4). The male average was heavily influenced by three individuals that sired more than six young each. One of these males, who successfully recruited eight young, was a founding individual in the first trial reintroduction in 2016 (siring 6–8 young; Wilson et al. 2020). The other two high‐output males (siring eight and 17 offspring, respectively) were born in the first generation. Removing these individuals reduced male averages to 2.68 offspring (median 2, ranging from 1 to −6) across an average of two litters (median 1, ranging 1–5).

Early cohorts likely faced lower rates of competition due to small founder cohorts (see Wilson et al. 2020, 2023). As the population approached density dependence the average body weights of individuals decreased (Wilson 2023). Our pedigree revealed that both the proportion of both males and females breeding successfully and the number of offspring recruited decreased markedly following the second breeding year (Figure S2, Table S6). We observed that litter size decreased over time from an average of 2.75 (2016) to 1.8 (2020), although this trend was not statistically significant (*R* = 0.82, *p* = 0.097). In contrast, the mean number of sires per litter remained stable (1.5 in both 2016 and 2019, *R* = 0.09; Table S6), indicating persistent polyandry despite declining recruitment. This suggests a preference for polyandry in the eastern quoll, as has been reported for the banded mongooses ( *Mungos mungo* ; Cant 2000) and agile *Antechinus* (Parrott et al. 2015).

Declining recruitment between 2017 and 2020 coincided with a severe drought that affected the eastern coast of Australia (BOM 2021) and would have created a high competition, resource‐limited environment. This is supported by declines in the number of offspring recruited per adult and the percentage of the adult population successfully breeding (Figure S2; Table S6), and also the lower average body weights we observed (Wilson 2023; Wilson et al. 2005). We propose that increasing competition between individuals for den or food resources may be more important to how successful a dam is in raising her young than her individual physiological traits. It is also possible, even likely, that random chance plays a large part in whether a dam successfully raised her offspring to independence. Continued monitoring of reproductive patterns during times of abundance would be needed to fully understand the selective pressures driving reproductive success. Another complicating factor may be that the individual, and litter, sample sizes that were available for our study may not have provided high enough resolution to detect patterns in the population. Ideally, each litter would be fully sampled while in the pouch and these genetics compared with individuals captured post‐dispersal to provide further insight into which individuals are successfully recruiting to the adult population, and the sampling completeness of our current dataset.

#### 3.1.4 Predictors of Reproductive Success

To determine predictors of eastern quoll reproductive success, we used generalised linear mixed models (GLMMs) to assess (1) the drivers of reproductive success or failure, and (2) the factors affecting the number of offspring recruited each year by successful breeders. Our models revealed that individual morphological traits (body weight, age, colour morph) strongly predicted reproductive success, explaining 60% of the variation within our dataset (Table 1, Tables S1–S3). Individual identity and year, while retained in the final model for completeness, had a negligible impact (Table 1, Tables S1–S3). Traits that did not significantly affect reproductive success included sex and individual heterozygosity. Upon inspection, we found model diagnostics (i.e., collinearity, overdispersion) indicated good fit and strong selective signals (residual variance approximated 0 on all models, Table 1), providing confidence that the trends we observed reflect real‐world patterns of breeding success, despite potential sampling limitations through having only collected post‐dispersal data.

**FIGURE 1:**
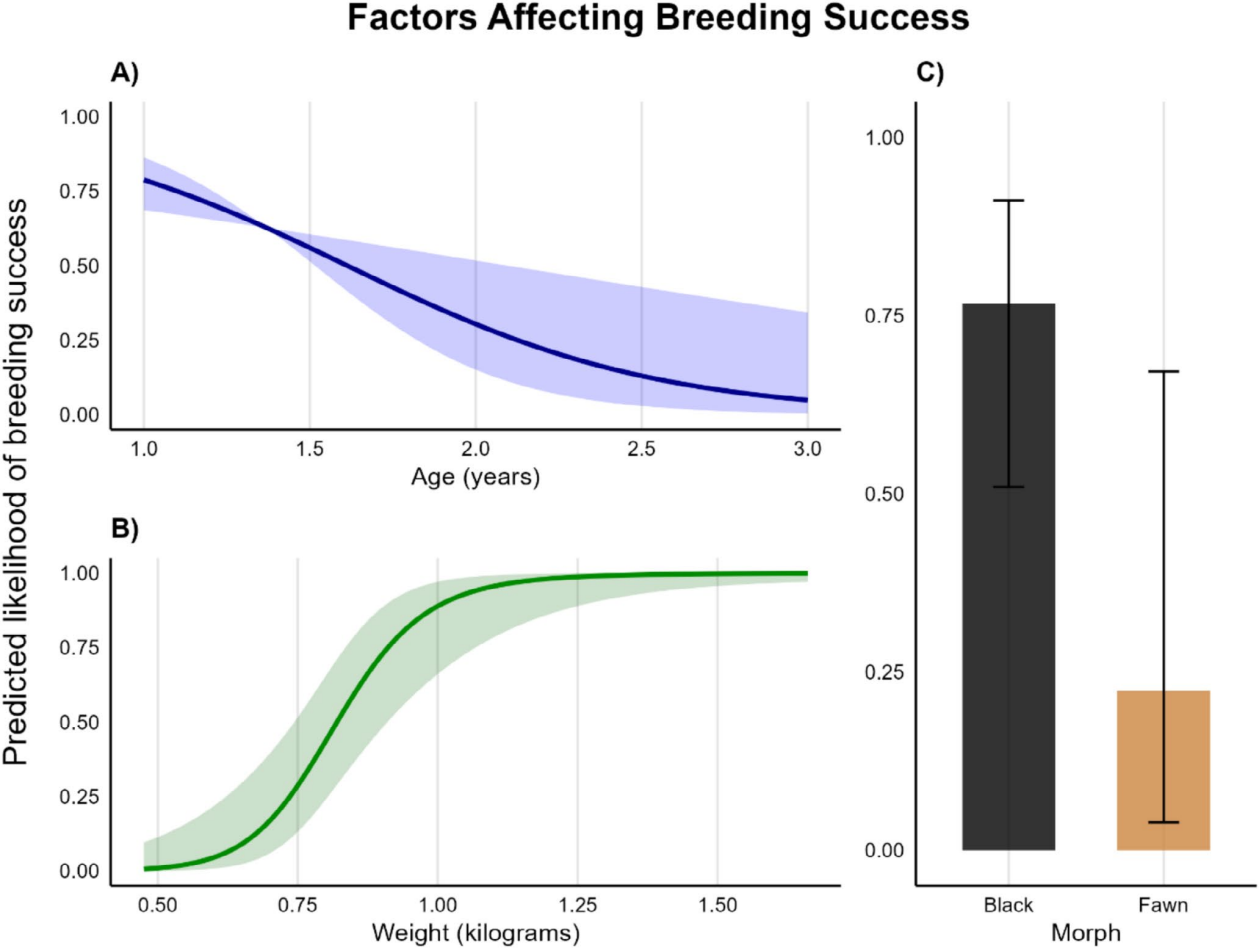
Predicted effects of individual traits on the likelihood of reproductive success* in the eastern quoll ( *Dasyurus viverrinus* ) population at Mulligans Flat Woodland Sanctuary, ACT, Australia. (A) The predicted likelihood of reproduction in each age class. Age 1 represents the first year following dispersal from the natal den, within which sexual maturity is reached (i.e., individuals within age class 1 are between 0 and 1 years old). Shaded areas represent a 95% confidence interval. (B) The predicted likelihood of reproduction relative to an individual's body weight in kilogrammes. Shaded areas represent a 95% confidence interval. (C) The predicted likelihood of reproduction for individuals displaying either of the two possible colour morphs for this species, black and fawn. Error bars represent standard error. *Note that reproductive success equates to recruitment of offspring into the population, as detected by capture and genetic sampling during regular population monitoring.

Our model data show age strongly reduced breeding probability: each additional year decreased the likelihood of recruitment by ~70% (OR: 0.26, *p* < 0.001; Table 1). Individuals over 2 years old were predicted to have a < 50% likelihood of reproducing at all (Figure 1). Higher body weight increased breeding success (heavy individuals were ~12 times more likely to recruit; OR = 12.24, *p* < 0.001; Table 1), but extremely heavy individuals performed poorly (OR = 0.50, *p* = 0.017; Table 1) likely reflecting age‐related decline rather than collinearity (males generally got heavier with age; Table S7). We used average weight data in our study, where multiple captures occurred in a year, and some resolution may have been lost. If an individual lost weight rapidly after the breeding season their average body weight would be lowered, which may be particularly likely for older males. The greatest within‐year difference recorded was approximately 800 g, a significant difference; however, most individuals had relatively consistent weights within a year, and we believe the effects of averaging to be minimal.

**FIGURE 2:**
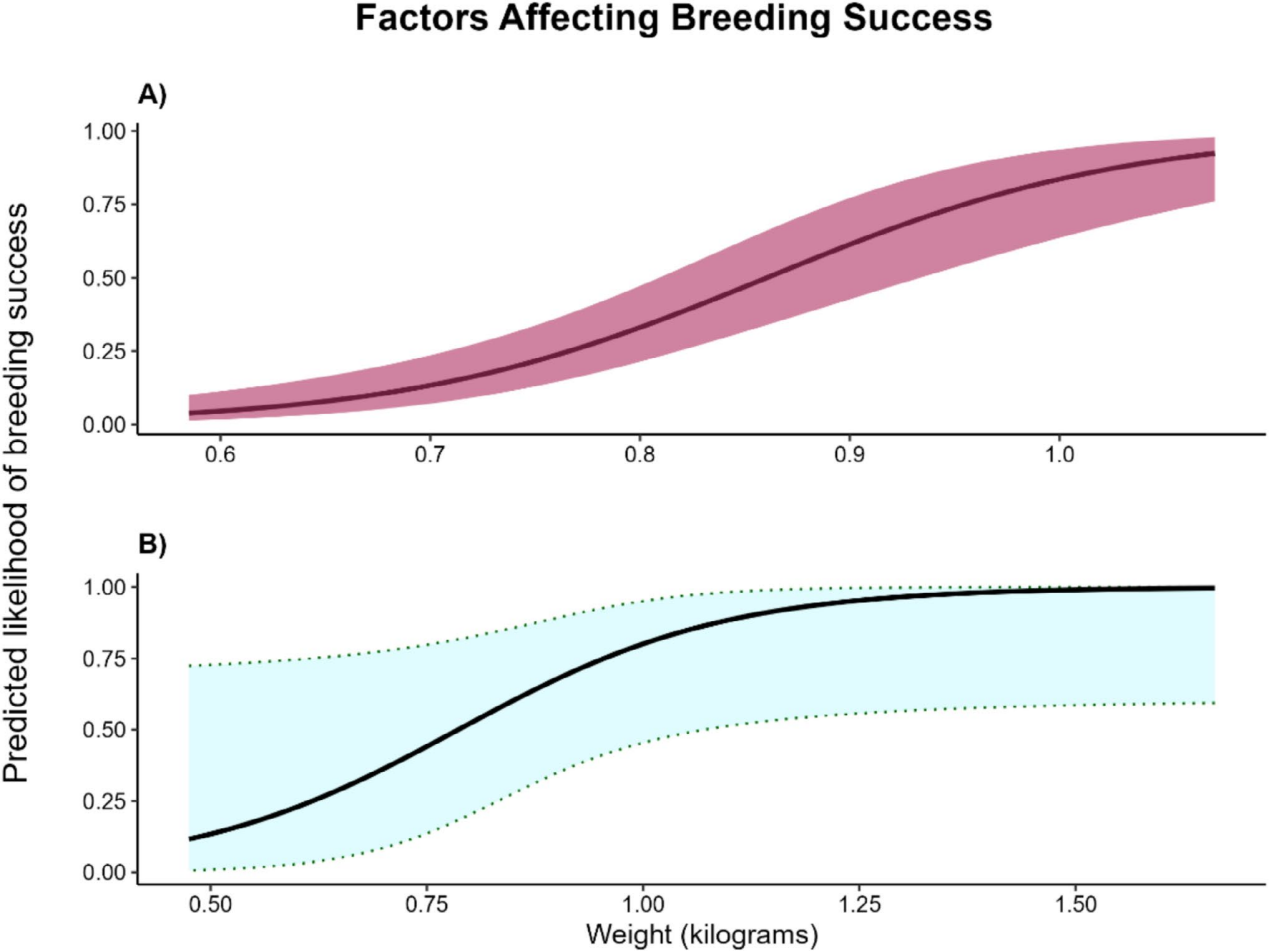
Predicted effect of individual weight (in kilogrammes) on reproductive success* of (A) male and (B) female eastern quolls ( *Dasyurus viverrinus* ) in the population at Mulligans Flat Woodland Sanctuary, ACT, Australia. Predictions based on results from binomial generalised linear mixed models conducted separately for each sex. Shaded areas represent a 95% confidence interval. * Note that reproductive success equates to recruitment of offspring into the population, as detected by capture and genetic sampling during population monitoring.

We initially attempted to incorporate sex as an interactive effect with weight and age, but this prevented model convergence (likely due to limited samples). Sex‐specific models confirmed that body weight significantly predicted breeding success for both males (OR 7.15, *p* = 0.052) and females (OR: 4.25, *p* = 0.005), though effect sizes and model explanatory power differed between sexes (Figure 2). Fixed effects explained 58% of variance in male reproductive success but negligible variance in female success (zero‐inflated model), suggesting different mechanisms may underlie male versus female breeding patterns. However, these sex‐specific results should be interpreted cautiously given limited sample sizes (19 males, 33 females). We did find that sanctuary‐born females were heavier on average (0.9 kg, maximum 1.6 kg) than those from Tasmania (averaging 0.7–0.8 kg, maximum 1.1 kg; Jones et al. 2001), while sanctuary‐born males were not. We posit that females within MFWS may have taken advantage of the increased resource availability within the landscape when the population was at a low density; the mammalian mesopredator niche was unfilled prior to the species' reintroduction. Despite males also having a seemingly low‐competition environment, we believe weight differences are likely attributable to the high stress of the breeding season that eastern quolls go through, which often affects male dasyurids more than females. Weight would, therefore, better reflect a female's ability to exploit resources than a male's in low‐competition environments in a relatively small area (485 ha).

Fawn coloured individuals within the MFWS population were predicted to have lower reproductive success than their dark‐morph counterparts (OR = 0.09, *p* = 0.003), which aligns with demographic monitoring data showing a population‐wide reduction in fawn‐morph frequency from 83% to 7% between 2016 and 2019. In the common brush‐tailed possum (*Trichosaurus vulpecula*), coat colour variation was found to be regulated by mutations within the agouti signalling protein (ASIP) coded for on chromosome 3—missense substitutions within the ASIP gene prevented ASIP production and resulted in blacker coloration of the fur (Bond et al. 2024). It has been posited by Bond et al. (2024) that dasyurids would be more prone to exhibiting these mutations because of the family's rapid evolution, which may be supported by Sauermann et al. (2025) assertion that loss‐of‐function mutations have occurred repeatedly within the marsupials and represent an important mechanism underpinning coat colour variation. Hartley et al. (2024) provided strong evidence that the ASIP gene is important in regulating colour morph in the eastern quoll, finding a deletion within the ASIP gene that they proposed underpins colour differences in both the eastern quoll and Tasmanian devil. Sauermann et al. (2025) propose that the selection for darker morph individuals may be a result of predator avoidance, because the eastern quoll is a nocturnal species. However, we can find little definitive evidence of whether colour morph, or the ASIP gene, is linked to other physiological or behavioural traits that may affect individual fitness. Drawing conclusions as to the cause of black‐morph dominance is not yet possible with much certainty and is beyond the scope of this article. Although it is unclear what is driving the difference in reproductive success between the colour morphs, our results show that there is a clear selective pressure negatively affecting fawn‐morph individuals within the MFWS population. There were 30 incidences of black‐morph male × black‐morph female crosses, 20 black × fawn, 13 fawn × black, and five fawn × fawn (Table S8). The reciprocal cross data in Table S8 indicates that the inheritance of colour morph is likely poly‐genic, and potentially sex‐linked (paternal morph may have more effect than maternal). However, more research is needed to understand the drivers of fitness, including morph inheritance, within eastern quoll populations.

#### 3.1.5 Predictors of Individual Fecundity

While body weight, age, and morph predicted whether individuals successfully reproduced, neither these traits nor individual heterozygosity or sex predicted how many offspring were produced by successful breeders (Table 1). The lack of fixed effect explanatory power (marginal *R* ^2^ ~ 0) but substantial individual variation (conditional *R* ^2^ = 0.515) suggests that unmeasured factors or random chance drive reproductive output (Table 1, Table S1). Potentially, additional resolution for both reproductive success and individual fecundity could be gained by further longitudinal sampling, including sampling of pouch young, which may reveal more nuanced patterns and the ability to investigate factors driving both breeding success and recruitment success.

### 3.2 Polyandry in the Dasyuridae

A meta‐analysis by Dobson et al. (2018) found that polyandrous species often exhibit multiple paternity in 25%–50% of all litters. They also posited that mating‐associated behaviours and ecological factors such as population density could prevent a positive, linear correlation between the number of sires and litter sizes. However, members of the Dasyuridae have been recorded as having up to 97.8% of litters with multiple sires (average 67.95%, Figure S1). Further, dasyurids tend to share similar life histories (semelparity, short maturation periods and lifespans, aggressive mating behaviour, and size dimorphism) but these vary along the spectrum for each trait. This variation means Dasyuridae can provide a useful case study for examining the drivers of multiple paternity frequency because the family displays highly variable rates of multiple paternity, is relatively well studied, and contains comparable life‐history strategies.

In our cross‐species comparison, the availability of information for the 11 dasyurid and two non‐dasyurid species was variable, despite being relatively well‐studied taxa. Breeding system studies for these species reported variable multiple paternity, ranging from 20% in the chuditch ( *Dasyurus geoffroii* ; Manning et al. 2022) to an exceptionally high 97.8% in the agile *Antechinus* ( *Antechinus agilis* ; Kraaijeveld‐Smit et al. 2002) (Figure S1). Our study found multiple paternity in 47.62% all eastern quoll litters, 62.50% of litters if only those with more than one offspring are assessed, or 87.51% if only the seven litters with four or more offspring are considered (Table 1). As such, the eastern quoll could have either the second or third lowest rate of multiple paternity within the family, or the third highest. The significant relationship we observed between larger litters and higher rates of multiple paternity (*R* = 0.53, *p* < 0.005; Table 1) suggests that if litter size is underestimated, multiple paternity frequency will be too. Given that we expected to have incomplete sampling of the MFWS population, and that we expected recruitment to be slightly higher than the frequency of single‐offspring litters would suggest, we have conservatively selected the intermediate multiple paternity frequency of 62.50% for the purposes of cross‐species comparisons. In this way, we balance the risk of underestimating multiple paternity frequency, without biasing our results based on small, incomplete sample sizes.

We also note that all other studies were based on pouch‐young sampling rather than post‐dispersal capture, as was done in our study. The lack of direct equivalency means that our measures of multiple paternity may be artificially lowered compared with the other studies (because our sampling was affected by post‐dispersal attrition). However, the patterns of multiple paternity within the comparison species are consistent with or without the eastern quoll data, indicating some level of robustness in the observed trends. We recommend that caution be taken if comparing our results to other study systems with a different sampling methodology but believe that our data offers a valuable contribution to the understanding of reproductive systems. Future studies should include both pouch and post‐dispersal sampling, so that patterns of both breeding and recruitment success can better be studied.

Despite likely being key feature of dasyurid reproductive strategies (Dickman 1994; Taggart et al. 2003), there is little sperm competition in the eastern quoll (Taggart et al. 2003), and high sperm competition in the agile *Antechinus* ( *Antechinus agilis* ; Shimmin et al. 2000). We theorise that differing levels of sperm competition may be linked to the relative prevalence of multiple paternity. Further investigation is required to determine whether sperm competition, or any other biological or behavioural characteristic, link shorter lifespans with higher rates of multiple paternity.

Our comparison revealed that neither sexual size dimorphism (*R* = 0.002, *p* = 0.884) or male‐to‐female aggression (*R* = 0.21, *p* = 0.15) impacted the rate of multiple paternity. This is inconsistent with findings from Wolff and Macdonald (2004), who found a need for aggression avoidance, including paternity confusion through matings with multiple males, can select for multiple paternity. Further, traits such as paternity confusion may be less relevant for taxa such as the dasyurids where males do not play a role in raising young. We acknowledge, however, that our qualitative categorisation of aggression from literature descriptions may have incorrectly assigned taxa to a group, or we may have missed intersex aggression cues relevant to the selection of multiple paternity. We recommend that future studies incorporate quantitative evaluations of aggression in species with multiple paternity. Our results also indicated that the ‘live fast–die young’ strategy of the assessed species may lead to increased multiple paternity. Shorter male lifespans correlated significantly with multiple paternity frequency across the assessed taxa (*R* = 0.35, *p* = 0.057), though female lifespans did not have a significant effect (*R* = 0.32, *p* = 0.109). It is possible that, where males of a species live through fewer breeding seasons, increased rates of multiple paternity are an evolutionary reflection of a highly competitive breeding system. There was no correlation between the frequency of multiple paternity and the maximum number of sires for a single litter (*R* = 0.03, *p* = 0.569).

We encountered difficulties sourcing comparable values for many ecological and behavioural characteristics that could feasibly be linked to the occurrence of multiple paternity, notably home range size, population density, and sperm competition or retention. We recommend future studies remedy these gaps in our understanding to enable more detailed analyses of these reproductive parameters.

## 4 Conclusions

Here, we revealed that the eastern quoll possesses a polyandrous breeding system with high rates of multiple paternity, furthering our understanding of the species' reproductive patterns and the key physiological traits that drive their reproductive fitness. We also expanded knowledge of the eastern quoll breeding system, finding that some males dominated the breeding pool in low‐competition environments and that females better exploited resource availability. Our cross‐species comparison revealed that male lifespan significantly affects the prevalence of multiple paternity litters in dasyurids. We recommend that future studies quantify traits such as copulatory frequency and duration, intersex aggression, and effective reproductive lifespan for inclusion in future breeding system studies. These studies should incorporate genomic sampling across a birth, within‐pouch, and post‐dispersal inclusive timeframe, to best tease out the complexities underpinning reproductive success.

## Author Contributions

**Brittany M. Brockett:** conceptualization (lead), data curation (lead), formal analysis (lead), investigation (lead), methodology (equal), project administration (lead), visualization (lead), writing – original draft (lead), writing – review and editing (lead). **Linda E. Neaves:** conceptualization (supporting), formal analysis (supporting), investigation (supporting), methodology (supporting), writing – original draft (supporting), writing – review and editing (equal). **Maldwyn J. Evans:** formal analysis (supporting), methodology (equal), writing – review and editing (supporting). **Iain J. Gordon:** conceptualization (supporting), writing – original draft (supporting), writing – review and editing (supporting). **Jennifer C. Pierson:** conceptualization (supporting), writing – original draft (supporting), writing – review and editing (supporting). **Belinda A. Wilson:** conceptualization (supporting), resources (equal), writing – review and editing (supporting). **Claire Wimpenny:** conceptualization (supporting), resources (supporting). **Adrian D. Manning:** conceptualization (supporting), funding acquisition (lead), project administration (supporting), resources (lead), writing – original draft (supporting), writing – review and editing (supporting).

## Funding

The data supporting this study was obtained for the Australian Research Council‐funded ‘Bringing Back Biodiversity’ project (LP140100209). BMB was funded by an ACT Government PhD Scholarship, an Australian Government Fee Offset Scholarship, and an ANU Supplementary Scholarship for part of the time in which this study occurred.

## Ethics Statement

Translocations were carried out under licences from the Tasmanian Department of Primary Industries, Parks, Water and Environment (permits TFA 16025 and 17,091, export licences 12,818/16 and 13,528/17), Victorian Department of Environment, Land, Water and Planning (permit 14,505,167), and Australian Capital Territory Directorate of Territory and Municipal Services (import licence L120161261). We thank DPIPWE for their support. The post‐reintroduction procedures were approved by the Australian National University Animal Experimentation Ethics Committee (protocol A2016/02).

## Conflicts of Interest

The authors declare no conflicts of interest.

## Supporting information


**Figure S1:** Phylogenetic relatedness and breeding system details for Australian marsupials. Phylogeny adapted from Westerman et al. (2016) and Krajewski et al. (2000). Values taken from sums using values presented by Dobson et al. (2018), are indicated by ^. **Litter size** provides the minimum and maximum number of possible offspring for a single litter, with the average number of offspring provided in (ellipses). Note two entries are present for *Dasyurus hallucatus* due to variation in different populations. **Litters sired multiply** indicated the percentage (%) of litters that have multiple fathers. **Sires** shows how many fathers have been observed in a single litter, with averages indicated in ellipses where available. **Miscellaneous notes on parentage** describes other aspects of maternity and paternity discussed by the relevant paper—notably, this includes paternity distribution within a litter. **Female: Male weight** is the percentage a female's weight is compared with a male (i.e., when males are larger than females, the value will be below 100%. A female of 10 g and a male of 20 g would result in 50%). **Life Span** describes the number of years a species lives in the wild. **Aggression** is a qualitative trait determined from descriptions in the literature: Low indicates that physical aggression was minimal, moderate that biting and some forced copulation occurs, and high indicates that biting is a regular feature of the species' breeding system, and may result in serious wounds. **(M)** indicates a male‐only trait, **(F)** a female‐only trait. Traits specific to the breeding season only are noted as **Br**, while traits that are specific to the non‐breeding season are indicated by **NB**. Where this study is reporting new information, it is **written in bold**. A* Indicates reproductive senescence, though individuals may survive beyond this point.
**Figure S2:** Trends in the percentage of adult eastern quolls ( *Dasyurus viverrinus* ) breeding successfully in the Mulligans Flat Woodland Sanctuary population. The *y*‐axis displays the observed breeding success of adult females and males (solid purple and blue lines, respectively). The *z*‐axis shows the number of known adult female and male eastern quolls within MFWS for a given year (dashed pink and blue lines, respectively). The ‘adult population’ of males and females was determined by assuming a lifespan of 4 years for males, as determined by age at first capture, and 5 years for females.
**Table S1:** Model selection table for a binomial generalised linear mixed model of eastern quoll ( *Dasyurus viverrinus* ) breeding success. The global model included weight (linear and quadratic terms), age, individual heterozygosity, sex, morph, and interactions between weight terms and age, with random effects of individual identity (id) and year. Models are ranked by AICc, with only models ΔAICc < 5 shown. A ‘+’ indicates the variable was included in the model. Continuous variables (weight, age, heterozygosity) were scaled (mean‐centred and standardised). The top‐ranked model (ΔAICc = 0, shown in **bold**) was selected for inference. All models converged successfully. Model selection was conducted using the dredge function in MuMIn package (Barton 2023). Random effects (individual id and year) were retained in all models.
**Table S2:** Model selection table for binomial generalised linear mixed model of female (top) and male (bottom) eastern quoll ( *Dasyurus viverrinus* ) breeding success. The global model included weight, age, individual heterozygosity, morph, with random effects of individual identity (id) and year. Models are ranked by AICc, with only models with ΔAICc < 5 shown. A ‘+’ indicates the variable was included in the model. Continuous variables (weight, age, heterozygosity) were scaled (mean‐centred and standardised). The top‐ranked model (ΔAICc = 0) was selected for inference for males, while the top three models were selected for averaging and subsequent inference for females. All models converged successfully. Model selection was conducted using the dredge function in MuMIn package (Barton 2023). Random effects (individual id and year) were retained in all models.
**Table S3:** Model selection table for Poisson‐based generalised linear mixed model of eastern quoll ( *Dasyurus viverrinus* ) fecundity. The global model included weight, age, individual heterozygosity, morph, with random effects of individual identity (id) and year. Models are ranked by AICc, with only models with ΔAICc < 5 shown. A ‘+’ indicates the variable was included in the model. Continuous variables (weight, age, heterozygosity) were scaled (mean‐centred and standardised). All models converged successfully. Model selection was conducted using the dredge function in MuMIn package (Barton 2023). Random effects (individual id and year) were retained in all models.
**Table S4:** Eastern quoll ( *Dasyurus viverrinus* ) pedigree for a population within Mulligans Flat Woodland Sanctuary (MFWS), ACT, Australia. Pedigree construction occurred in Colony v 2.0.6.6, and was informed by 1745 single‐nucleotide polymorphisms and demographic data obtained through long‐term monitoring of the MFWS population. Birth year for each ‘Offspring’ born within MFWS was assigned based on age at first capture, and impossible parents (i.e., individuals not yet born based on age at first capture, individuals not yet translocated into the MFWS population, or individuals that would have died due to old age) excluded from consideration. Where data were available, offspring were assigned to their known mother. The pedigree‐assigned ‘Mother’ and ‘Father’ for each MFWS individual were then recorded, and for each year (i.e., 2016, 2017, etc.) all offspring of a single female were grouped into ‘Litters’.
**Table S5:** Comparison of litter size and multiple paternity within the eastern quoll ( *Dasyurus viverrinus* ) population at Mulligans Flat Woodland Sanctuary (MFWS), ACT, Australia. Data derived from a pedigree constructed in Colony v 2.0.6.6. Pedigree construction was informed by 1745 single‐nucleotide polymorphisms and demographic data obtained through long‐term monitoring of the MFWS population. ‘Litter size’ represents various subsets of the total dataset based on the minimum number of offspring in a litter, and ‘Count’ displays the number of litters that were reconstructed in the pedigree. The frequency of multiple paternity is represented by the ‘% litters with multiple paternity’, being a count of how many litters within the subset had multiple fathers assigned in the pedigree. The ‘Mean number of sires’ presents the average number of fathers assigned to litters within the pedigree.
**Table S6:** Reproductive patterns in a population of reintroduced eastern quolls ( *Dasyurus viverrinus* ) at Mulligans Flat Woodland Sanctuary (MFWS), ACT, Australia. Parentage data obtained from a pedigree constructed in Colony v 2.0.6.6, informed by 1745 single‐nucleotide polymorphisms and demographic data obtained through long‐term monitoring of the MFWS population. *R* ^2^ and *p*‐values obtained through simple linear regression.
**Table S7:** Model diagnostic tables for investigation into eastern quoll ( *Dasyurus viverrinus* ) breeding systems. Results of the check_collinearity and check_overdispersion functions of the *performance* package v0.15.3, in R v4.5.0.
**Table S8:** Reciprocal cross data from eastern quolls ( *Dasyurus viverrinus* ) born into the Mulligans Flat Woodland Sanctuary, Canberra ACT, between 2017 and 2020. Data derived using 1745 single‐nucleotide polymorphisms and long‐term demographic data to reconstruct a pedigree in Colony v 2.0.6.6.

## Data Availability

The data that support the findings of this study, including SNP reads and analysis pathways, are openly available in the ANU Data Commons repository at http://doi.org/10.25911/50fr‐x765.
