# Supplementary material for: Factors Affecting Multiple Paternity: Insights From the Eastern Quoll (Dasyurus viverrinus)
Source: Ecol Evol. 2026 Apr 30;16(5):e73515. doi: 10.1002/ece3.73515 (PMC13130336; doi:10.1002/ece3.73515)
Supplement: Supplementary file 1 — Figure S1: Phylogenetic relatedness and breeding system details for Australian marsupials. Phylogeny adapted from Westerman et al. (2016) and Krajewski et al. (2000). Values taken from sums using values presented by Dobson et al. (2018), are indicated by ^. Litter size provides the minimum and maximum number of possible offspring for a single litter, with the average number of offspring provided in (ellipses). Note two entries are present for Dasyurus hallucatus due to variation in different populations. Litters sired multiply indicated the percentage (%) of litters that have multiple fathers. Sires shows how many fathers have been observed in a single litter, with averages indicated in ellipses where available. Miscellaneous notes on parentage describes other aspects of maternity and paternity discussed by the relevant paper—notably, this includes paternity distribution within a litter. Female: Male weight is the percentage a female's weight is compared with a male (i.e., when males are larger than females, the value will be below 100%. A female of 10 g and a male of 20 g would result in 50%). Life Span describes the number of years a species lives in the wild. Aggression is a qualitative trait determined from descriptions in the literature: Low indicates that physical aggression was minimal, moderate that biting and some forced copulation occurs, and high indicates that biting is a regular feature of the species' breeding system, and may result in serious wounds. (M) indicates a male‐only trait, (F) a female‐only trait. Traits specific to the breeding season only are noted as Br, while traits that are specific to the non‐breeding season are indicated by NB. Where this study is reporting new information, it is written in bold. A* Indicates reproductive senescence, though individuals may survive beyond this point. Figure S2: Trends in the percentage of adult eastern quolls ( Dasyurus viverrinus ) breeding successfully in the Mulligans Flat Woodland Sanctuary populat [file ECE3-16-e73515-s001.zip › 2026-01-10 - Factors affecting multiple paternity - Supplementary Information.docx]

## Supplementary information

Supplementary Table 1. Model selection table for a binomial generalised linear mixed-model of eastern quoll (*Dasyurus viverrinus*) breeding success. The global model included weight (linear and quadratic terms), age, individual heterozygosity, sex, morph, and interactions between weight terms and age, with random effects of individual identity (id) and year. Models are ranked by AICc, with only models ΔAICc < 5 shown. A "+" indicates the variable was included in the model. Continuous variables (weight, age, heterozygosity) were scaled (mean-centered and standardised). The top-ranked model (ΔAICc = 0, shown in **bold**) was selected for inference. All models converged successfully. Model selection was conducted using the dredge function in MuMIn package (Barton, 2023). Random effects (individual id and year) were retained in all models.


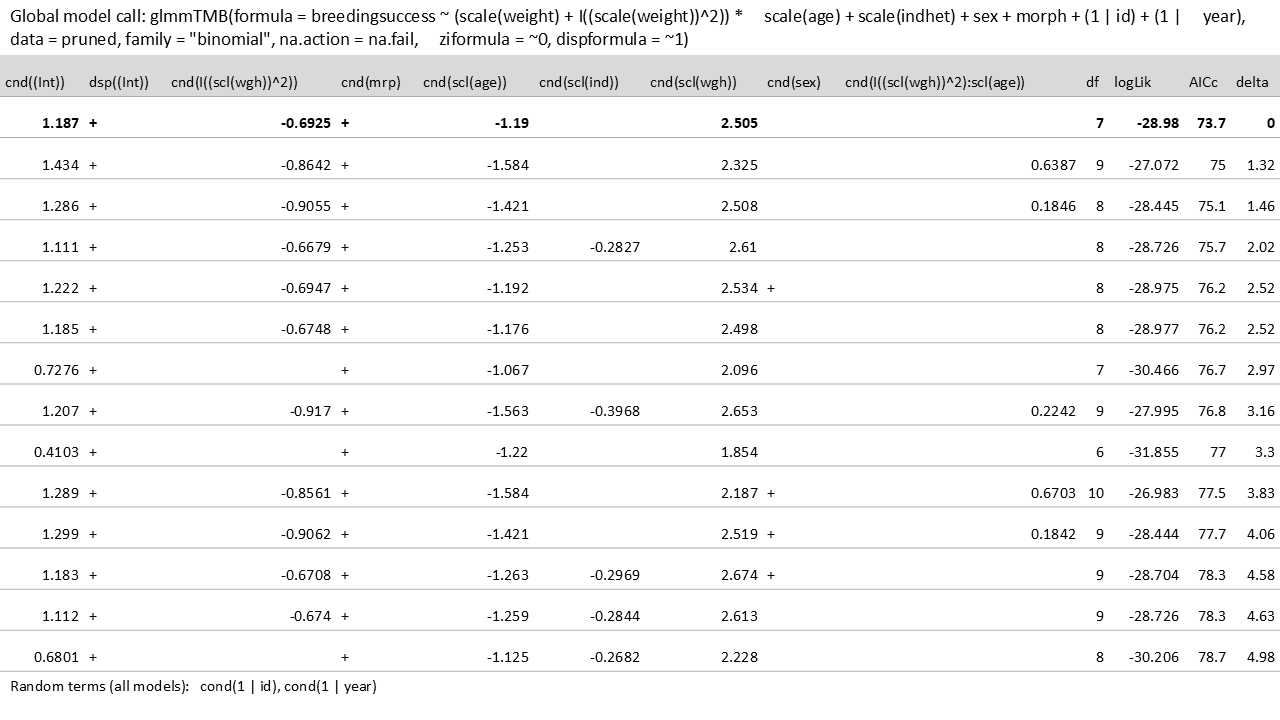


Supplementary Table 2. Model selection table for binomial generalised linear mixed-model of female (top) and male (bottom) eastern quoll (*Dasyurus viverrinus*) breeding success. The global model included weight, age, individual heterozygosity, morph, with random effects of individual identity (id) and year. Models are ranked by AICc, with only models with ΔAICc < 5 shown. A "+" indicates the variable was included in the model. Continuous variables (weight, age, heterozygosity) were scaled (mean-centered and standardised). The top-ranked model (ΔAICc = 0) was selected for inference for males, while the top three models were selected for averaging and subsequent inference for females. All models converged successfully. Model selection was conducted using the dredge function in MuMIn package (Barton, 2023). Random effects (individual id and year) were retained in all models.


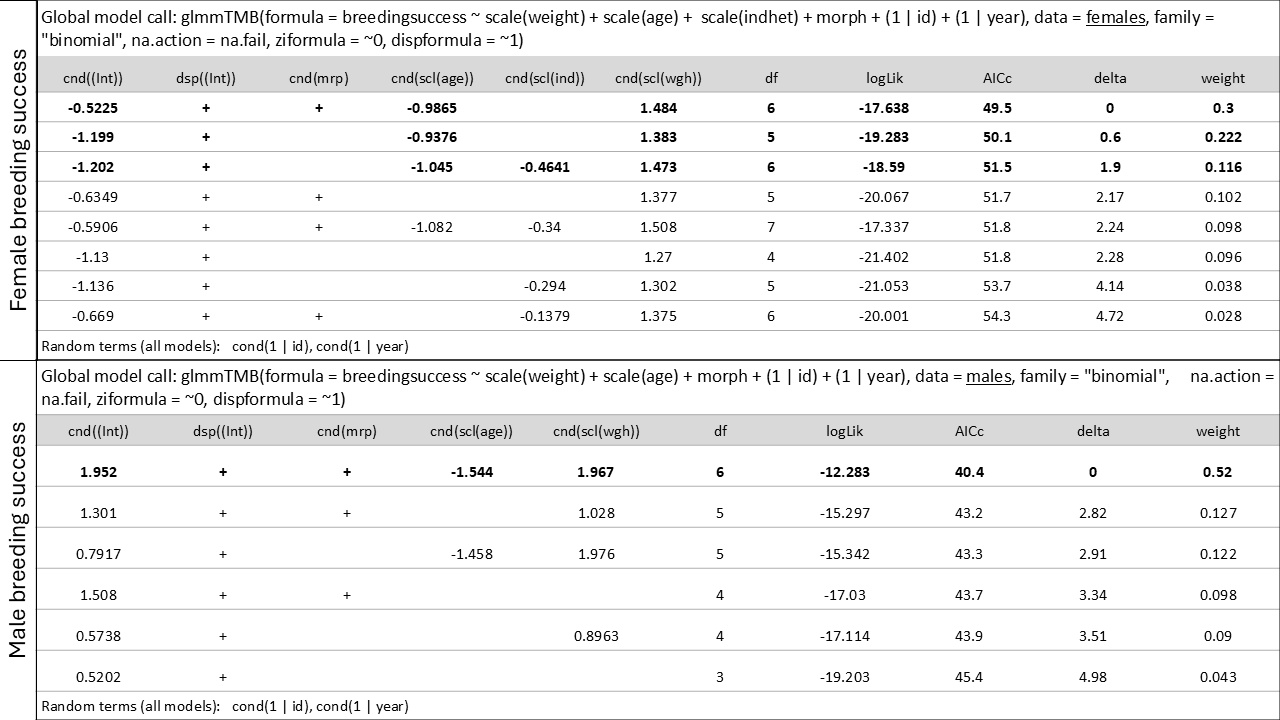


Supplementary Table 3. Model selection table for poisson-based generalised linear mixed-model of eastern quoll (*Dasyurus viverrinus*) fecundity. The global model included weight, age, individual heterozygosity, morph, with random effects of individual identity (id) and year. Models are ranked by AICc, with only models with ΔAICc < 5 shown. A "+" indicates the variable was included in the model. Continuous variables (weight, age, heterozygosity) were scaled (mean-centered and standardised). All models converged successfully. Model selection was conducted using the dredge function in MuMIn package (Barton, 2023). Random effects (individual id and year) were retained in all models.
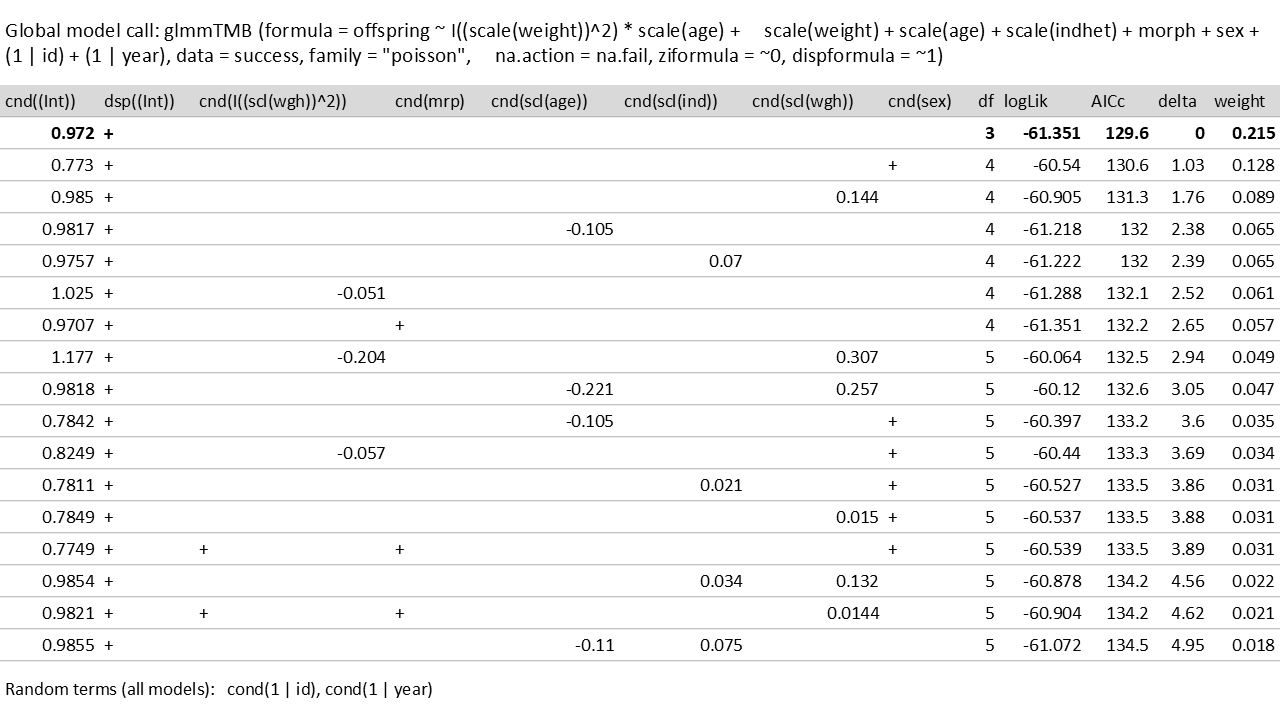


*
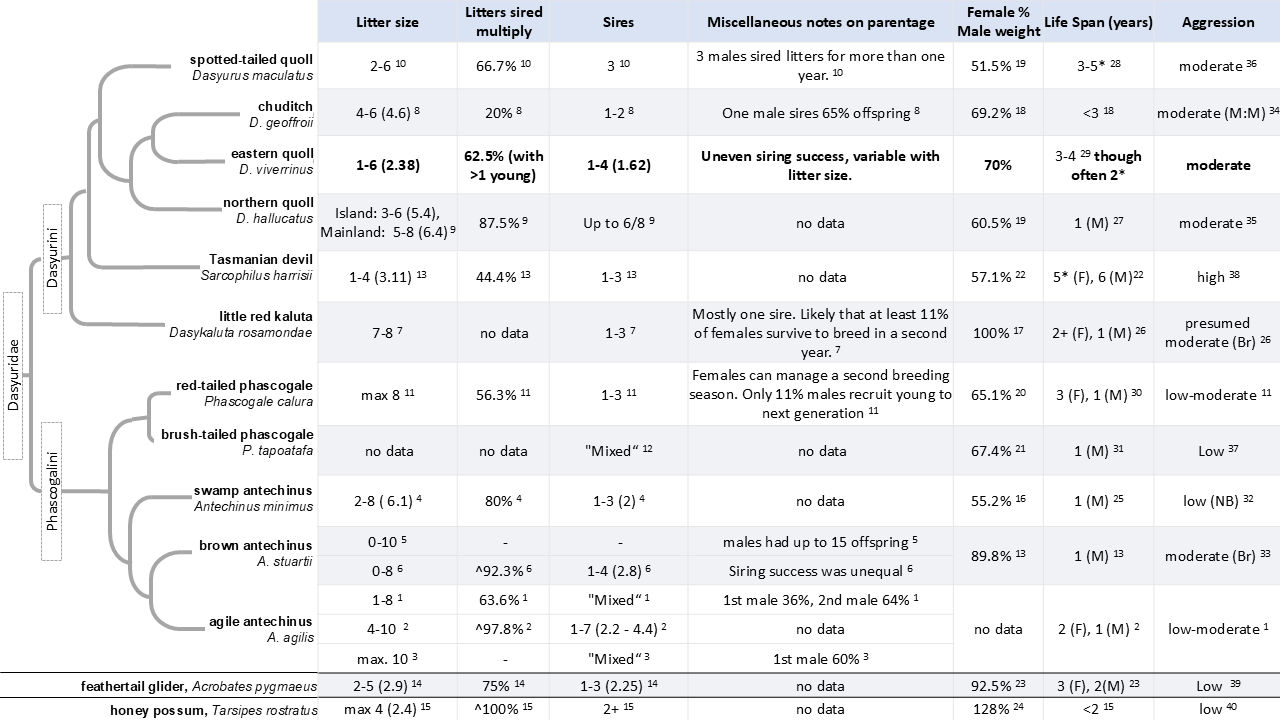
*

Supplementary Figure 1. Phylogenetic relatedness and breeding system details for Australian marsupials. Phylogeny adapted from Westerman et al. (2016) and Krajewski et al. (2000). Values taken from sums using values presented by Dobson et al (2018), are indicated by ^. **Litter size** provides the minimum and maximum number of possible offspring for a single litter, with the average number of offspring provided in (ellipses). Note two entries are present for Dasyurus hallucatus due to variation in different populations. **Litters sired multiply** indicated the percentage (%) of litters that have multiple fathers. **Sires** shows how many fathers have been observed in a single litter, with averages indicated in ellipses where available. **Miscellaneous notes on parentage** describes other aspects of maternity and paternity discussed by the relevant paper – notably, this includes paternity distribution within a litter. **Female : Male weight** is the percentage a female’s weight is compared to a male (i.e. when males are larger than females, the value will be below 100%. A female of 10g and a male of 20g would result in 50%). **Life Span** describes the number of years a species lives in the wild. **Aggression** is a qualitative trait determined from descriptions in the literature: Low indicates that physical aggression was minimal, moderate that biting and some forced copulation occurs, and high indicates that biting is a regular feature of the species’ breeding system, and may result in serious wounds. **(M)** indicates a male-only trait, **(F)** a female-only trait. Traits specific to the breeding season only are noted as **Br**, while traits that are specific to the non-breeding season are indicated by **NB**. Where this study is reporting new information, it is **written in bold**. A ***** Indicates reproductive senescence, though individuals may survive beyond this point.

**Source indicated by superscript:** **1 (Shimmin, Taggart, & Temple‐Smith, 2002)**; **2 (Kraaijeveld-Smit, Ward, & Temple-Smith, 2002)**; **3 (Fisher, Double, & Moore, 2006)**; **4 (M. G. Sale, Kraaijeveld-Smit, & Arnould, 2013)**; **5 (Fisher, Double, Blomberg, Jennions, & Cockburn, 2006)**; **6 (Holleley, Dickman, Crowther, & Oldroyd, 2006)**; **7 (Hayes et al., 2019)**; **8 (Manning, Austin, Moseby, & Jensen, 2022)**; **9 (Chan, Dunlop, & Spencer, 2020)**; **10 (Glen, Cardoso, Dickman, & Firestone, 2009)**; **11 (W. Foster, 2008)**; **12** **Millis, 1995** (in **(W. Foster, 2008; M. G. Sale, Kraaijeveld-Smit, & Arnould, 2009)**); **13 (Russell, 2018)**; **14 (Parrott, Ward, & Taggart, 2005)**; **15 (Wooller, Richardson, Garavanta, Saffer, & Bryant, 2000)**; **16 (Michael G. Sale, Wilson, & Arnould, 2008)**; **17 (Kortner, Rojas, & Geiser, 2010)**; **18 (Serena & Soderquist, 1988)**; **19 (Cooper & Withers, 2010)**; **20 (W. K. Foster & Taggart, 2008)**; **21 (Todd R. Soderquist, 1995)**; **22 (Guiler, 1970)**; **23 (Ward, 1990)**; **24 (Bryant, 2004)**; **25 (M. G. Sale et al., 2009)**; **26 (Woolley, 1991)**; **27 (Oakwood, 1997)**; **28 (DELWP, 2016)**; **29 (Godsell, 1983)**; **30 (Bradley, 2009)**; **31** **Cuttle, 1982** (in **(Todd R. Soderquist, 1995)**); **32 (M. G. SALE & ARNOULD, 2009)**; **33 (Braithwaite, 1974)**; **34 (SERENA & SODERQUIST, 1989)**; **35 (Oakwood, 2002)**; **36 (Belcher & Darrant, 2006)**; **37 (T. R. Soderquist & Ealey, 1994)**; **38 (Hamede, McCallum, & Jones, 2008)**; **39 (Kirk, Smith, & Agnew, 2000)**; **40 (Wooller et al., 2000)**.

Supplementary Table 4. Eastern quoll (*Dasyurus viverrinus*) pedigree for a population within Mulligans Flat Woodland Sanctuary (MFWS), ACT, Australia. Pedigree construction occurred in Colony v 2.0.6.6, and was informed by 1745 single nucleotide polymorphisms and demographic data obtained through long term monitoring of the MFWS population. Birth year for each “Offspring” born within MFWS was assigned based on age at first capture, and impossible parents (i.e. individuals not yet born based on age at first capture, individuals not yet translocated into the MFWS population, or individuals that would have dies due to old age) excluded from consideration. Where data was available, offspring were assigned to their known mother. The pedigree-assigned “Mother” and “Father” for each MFWS individual were then recorded, and for each year (i.e. 2016, 2017, etc) all offspring of a single female were grouped into “Litters”.


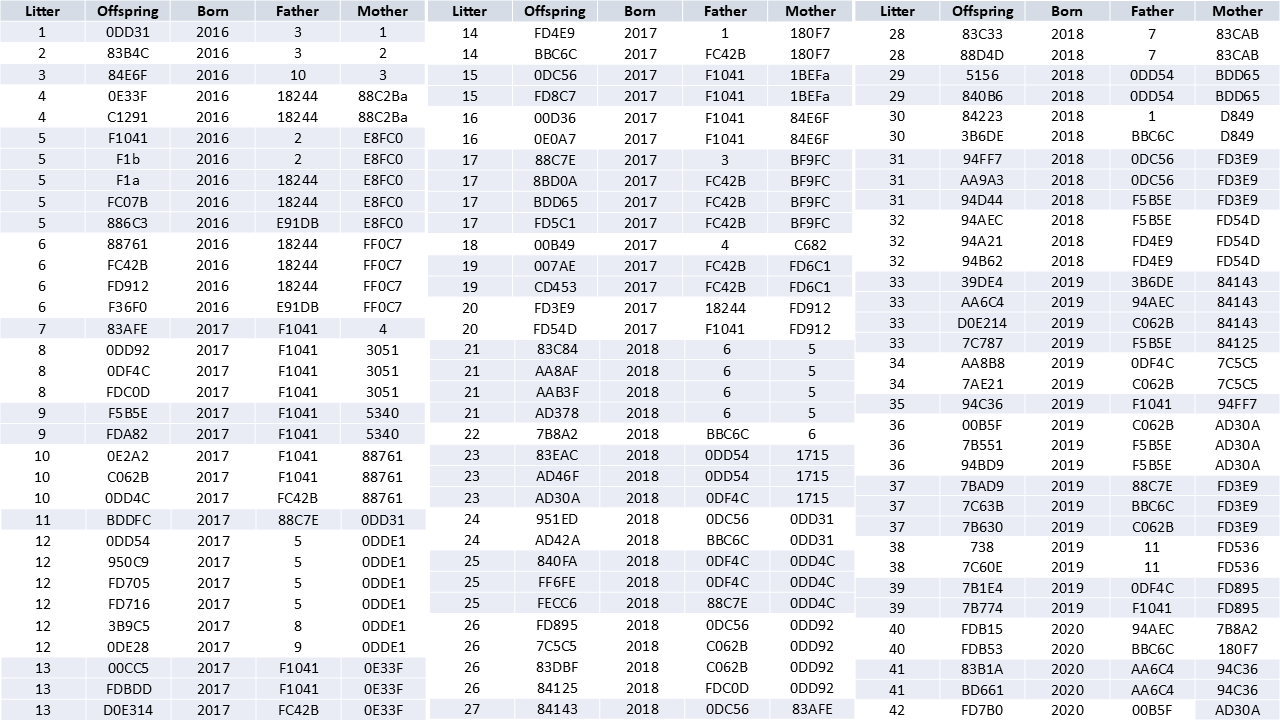


Supplementary Table 5. Comparison of litter size and multiple paternity within the eastern quoll (*Dasyurus viverrinus*) population at Mulligans Flat Woodland Sanctuary (MFWS), ACT, Australia. Data derived from a pedigree constructed in Colony v 2.0.6.6. Pedigree construction was informed by 1745 single nucleotide polymorphisms and demographic data obtained through long term monitoring of the MFWS population. “Litter size” represents various subsets of the total dataset based on the minimum number of offspring in a litter, and “Count” displays the number of litters that were reconstructed in the pedigree. The frequency of multiple paternity is represented by the “% litters with multiple paternity”, being a count of how many litters within the subset had multiple fathers assigned in the pedigree. The “Mean number of sires” presents the average number of fathers assigned to litters within the pedigree.


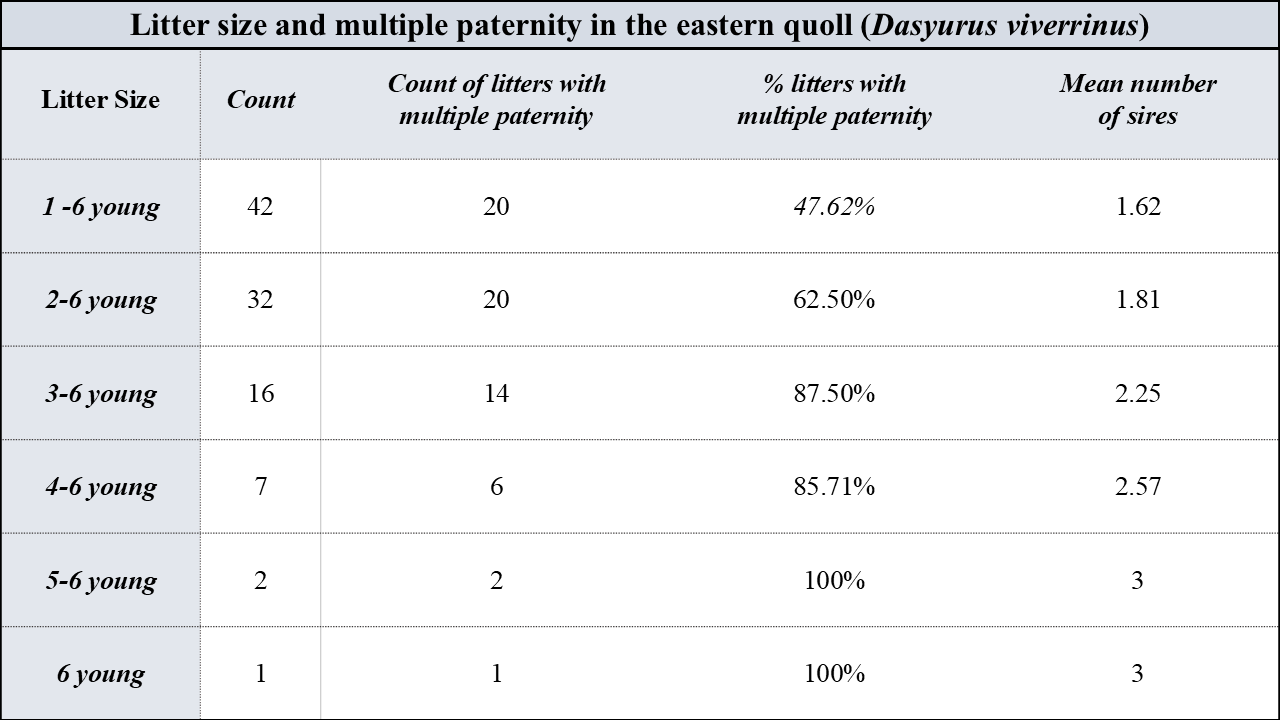


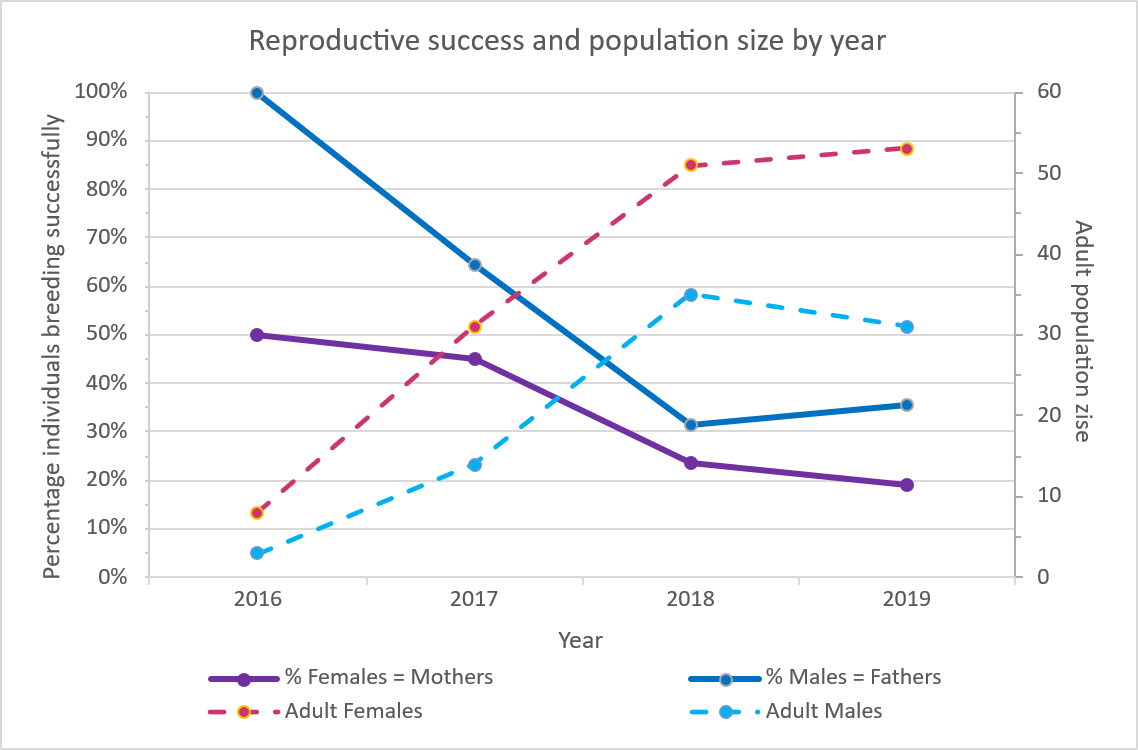


Supplementary Figure 2. Trends in the percentage of adult eastern quolls (Dasyurus viverrinus) breeding successfully in the Mulligans Flat Woodland Sanctuary population. The y-axis displays the observed breeding success of adult females and males (solid purple and blue lines, respectively). The z-axis shows the number of known adult female and male eastern quolls within MFWS for a given year (dashed pink and blue lines, respectively). The “adult population” of males and females was determined by assuming a lifespan of 4 years for males, as determined by age at first capture, and 5 years for females.

Supplementary Table 6. Reproductive patterns in a population of reintroduced eastern quolls (*Dasyurus viverrinus*) at Mulligans Flat Woodland Sanctuary (MFWS), ACT, Australia. Parentage data obtained from a pedigree constructed in Colony v 2.0.6.6, informed by 1745 single nucleotide polymorphisms and demographic data obtained through long term monitoring of the MFWS population. R^2^ and p values obtained through simple linear regression.


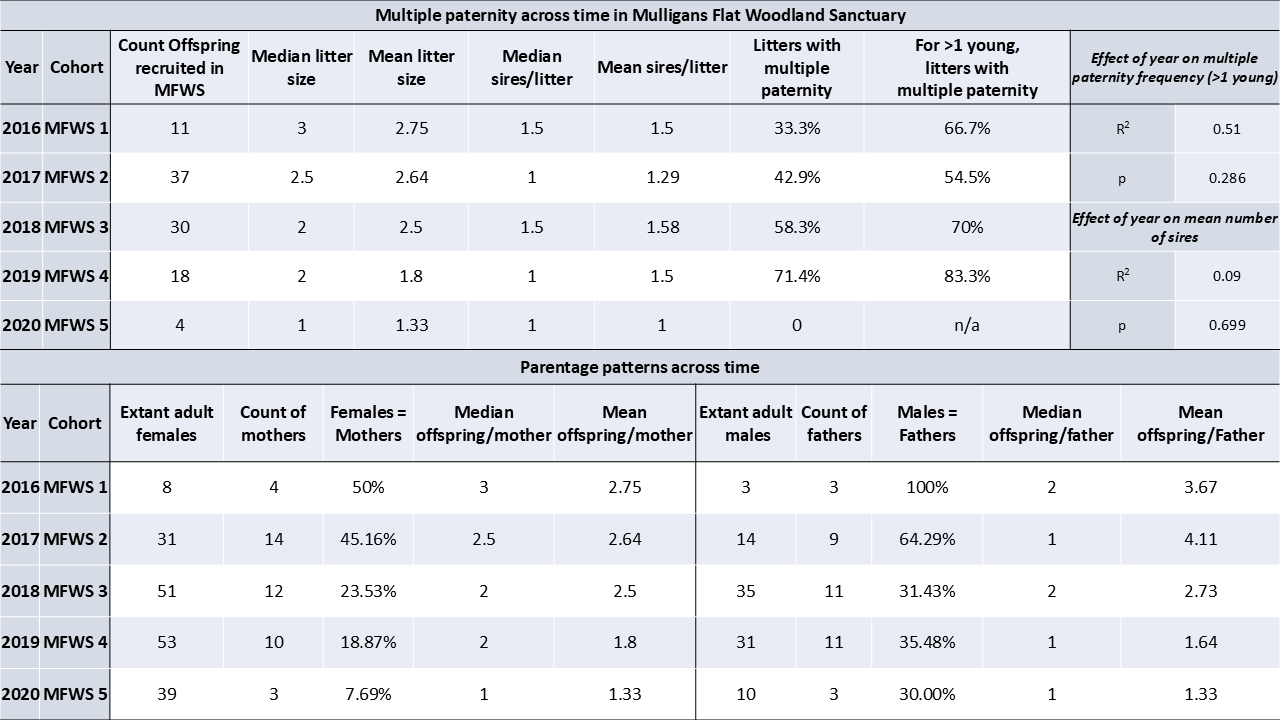


Supplementary Table 7. Model diagnostic tables for investigation into eastern quoll (*Dasyurus viverrinus*) breeding systems. Results of the check_collinearity and check_overdispersion functions of the *performance* package v0.15.3), in R v4.5.0.
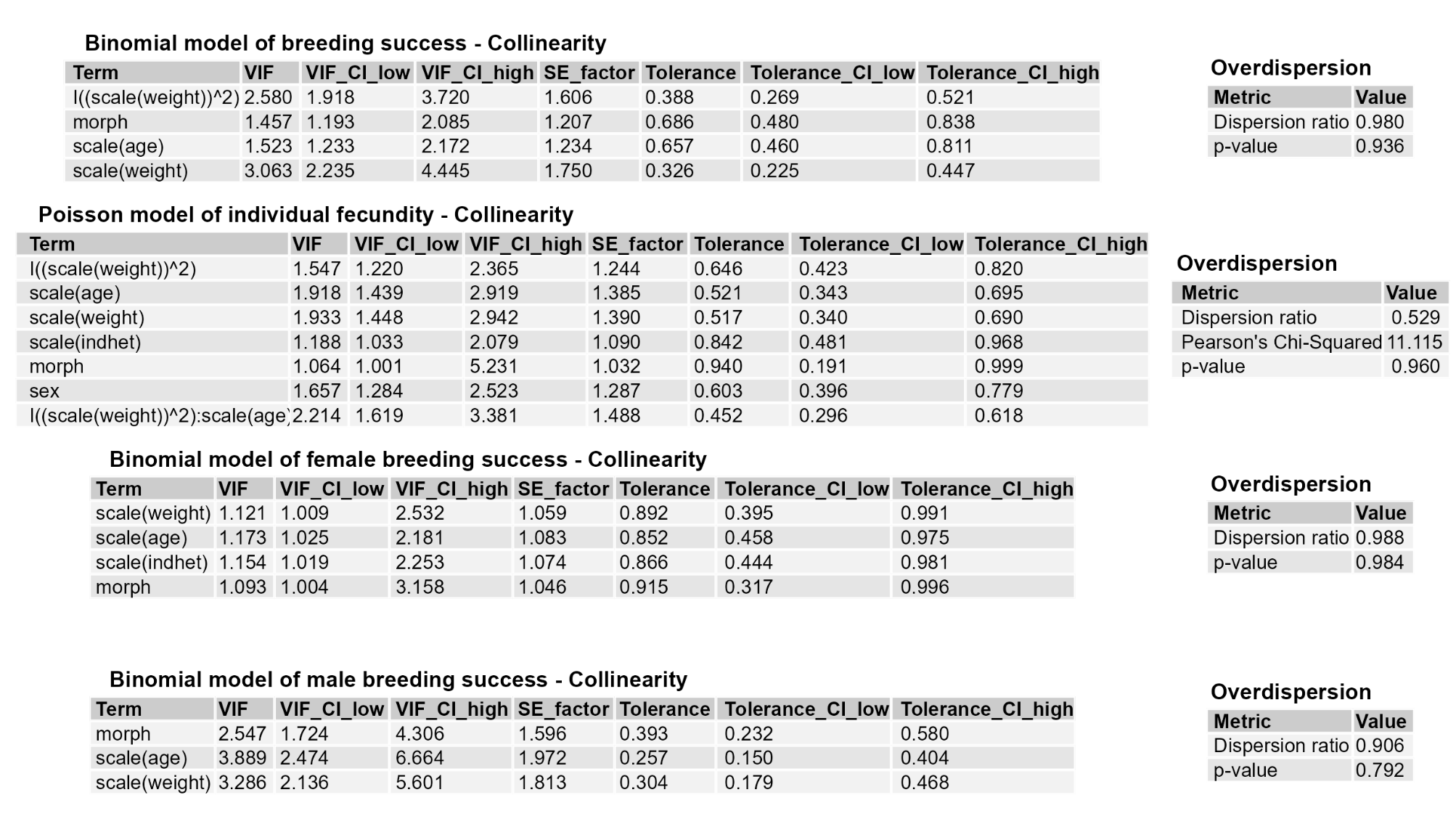


Supplementary Table 8. Reciprocal cross data from eastern quolls (Dasyurus viverrinus) born into the Mulligans Flat Woodland Sanctuary, Canberra ACT, between 2017 and 2020. Data derived using 1745 single-nucleotide polymorphisms and long-term demographic data to reconstruct a pedigree in Colony v 2.0.6.6

| Parental cross | | Count | | | % Offspring Black | % Offspring Fawn |
| --- | --- | --- | --- | --- | --- | --- |
| Father | Mother | Incidences | Black Offspring | Fawn Offspring |  |  |
| Black | Black | 30 | 27 | 3 | 90 | 10 |
| Fawn | Black | 20 | 9 | 11 | 45 | 55 |
| Black | Fawn | 13 | 4 | 9 | 30.8 | 69.2 |
| Fawn | Fawn | 5 | 2 | 3 | 40 | 60 |

References

Belcher, C. A., & Darrant, J. P. (2006). Home range and spatial organization of the marsupial carnivore, Dasyurus maculatus maculatus (Marsupialia: Dasyuridae) in south‐eastern Australia. *Journal of Zoology, 262*(3), 271-280. doi:10.1017/s0952836903004631

Bradley, A. J. (2009). Reproduction and life history in the red‐tailed phascogale, *Phascogale calura* (Marsupialia: Dasyuridae): the adaptive‐stress senescence hypothesis. *Journal of Zoology, 241*(4), 739-755. doi:10.1111/j.1469-7998.1997.tb05745.x

Braithwaite, R. W. (1974). Behavioural Changes associated with the Population Cycle of Antechinus stuavtii (Marsupialia). *Australian Journal of Zoology, 22*, 45-62.

Bryant, K. A. (2004). *The mating system and reproduction in the honey possum, Tarsipes rostratus: a life-history and genetical perspective.* (Doctorate of Philosophy). Murdoch University,

Chan, R., Dunlop, J., & Spencer, P. B. S. (2020). Highly promiscuous paternity in mainland and island populations of the endangered Northern Quoll. *Journal of Zoology, 310*, 210-220. doi:10.1111/jzo.12745

Cooper, C. E., & Withers, P. C. (2010). Comparative physiology of Australian quolls (Dasyurus; Marsupialia). *J Comp Physiol B, 180*(6), 857-868. doi:10.1007/s00360-010-0452-3

DELWP. (2016). *National Recovery Plan for the Spotted-tailed Quoll Dasyurus maculatus*. Canberra: Australian Government Department of the Environment Retrieved from <https://www.dcceew.gov.au/environment/biodiversity/threatened/recovery-plans/spotted-tailed-quoll>

Fisher, D. O., Double, M. C., Blomberg, S. P., Jennions, M. D., & Cockburn, A. (2006). Post-mating sexual selection increases lifetime fitness of polyandrous females in the wild. *Nature, 444*(7115), 89-92. doi:10.1038/nature05206

Fisher, D. O., Double, M. C., & Moore, B. D. (2006). Number of mates and timing of mating affect offspring growth in the small marsupial Antechinus agilis. *Animal Behaviour, 71*(2), 289-297. doi:10.1016/j.anbehav.2005.03.041

Foster, W. (2008). *Reproductive strategies of the red-tailed phascogale (Phascogale calura).* (Doctor of Philosophy). The University of Adelaide,

Foster, W. K., & Taggart, D. A. (2008). Gender and parental influences on the growth of a sexually dimorphic carnivorous marsupial. *Journal of Zoology, 275*(3), 221-228. doi:10.1111/j.1469-7998.2008.00429.x

Glen, A. S., Cardoso, M. J., Dickman, C. R., & Firestone, K. B. (2009). Who’s your daddy? Paternity testing reveals promiscuity and multiple paternity in the carnivorous marsupial Dasyurus maculatus (Marsupialia: Dasyuridae). *Biological Journal of the Linnean Society, 96*, 1-7. doi:<https://doi.org/10.1111/j.1095-8312.2008.01094.x>

Godsell, J. (1983). *ECOLOGY OF THE EASTERN QUOLL DASYURUS VIVERRINUS, (DASYURIDAE : MARSUPIALIA).* (Doctorate of Philosophy). Australian National University, Canberra.

Guiler, E. R. (1970). OBSERVATIONS ON THE TASMANIAN DEVIL, *SARCOPHILUS HARRISII* (MARSUPIALIA : DASYURIDAE) II. REPRODUCTION, BREEDING, AND GROWTH OF POUCH YOUNG. *Australian Journal of Zoology, 18*, 63-70.

Hamede, R. K., McCallum, H., & Jones, M. (2008). Seasonal, demographic and density-related patterns of contact between Tasmanian devils (Sarcophilus harrisii): Implications for transmission of devil facial tumour disease. *Austral Ecology, 33*(5), 614-622. doi:10.1111/j.1442-9993.2007.01827.x

Hayes, G. L. T., Simmons, L. W., Dugand, R. J., Mills, H. R., Roberts, J. D., Tomkins, J. L., & Fisher, D. O. (2019). Male semelparity and multiple paternity confirmed in an arid‐zone dasyurid. *Journal of Zoology, 308*(4), 266-273. doi:10.1111/jzo.12672

Holleley, C. E., Dickman, C. R., Crowther, M. S., & Oldroyd, B. P. (2006). Size breeds success: multiple paternity, multivariate selection and male semelparity in a small marsupial, Antechinus stuartii. *Mol Ecol, 15*(11), 3439-3448. doi:10.1111/j.1365-294X.2006.03001.x

Kirk, J., Smith, G. C., & Agnew, G. (2000). Trial radio-tracking of feathertail gliders Acrobates pygmaeus. *Australian Mammalogy, 22*, 129-131.

Kortner, G., Rojas, A. D., & Geiser, F. (2010). Thermal biology, torpor use and activity patterns of a small diurnal marsupial from a tropical desert: sexual differences. *J Comp Physiol B, 180*(6), 869-876. doi:10.1007/s00360-010-0459-9

Kraaijeveld-Smit, F., Ward, S., & Temple-Smith, P. (2002). Multiple paternity in a field population of a small carnivorous marsupial, the agile antechinus, Antechinus agilis. *Behavioral Ecology and Sociobiology, 52*(1), 84-91. doi:10.1007/s00265-002-0485-z

Manning, T. P., Austin, J. J., Moseby, K. E., & Jensen, M. A. (2022). Skewed paternity impacts genetic diversity in a small reintroduced population of western quolls (Dasyurus geoffroii) - Supplementary Material. *Australian Mammalogy*. doi:10.1071/am22012

Oakwood, M. (1997). *The Ecology of the Northern Quoll, Dasyurus hallucatus.* (Doctorate of Philosophy Thesis (PhD)). The Australian National University, Canberra.

Oakwood, M. (2002). Spatial and social organization of a carnivorous marsupial Dasyurus hallucatus (Marsupialia: Dasyuridae). *Journal of Zoology, 257*(2), 237-248. doi:10.1017/s0952836902000833

Parrott, M. L., Ward, S. J., & Taggart, D. A. (2005). Multiple paternity and communal maternal care in the feathertail glider (Acrobates pygmaeus). *Australian Journal of Zoology, 53*, 79-85.

Russell, T. C. (2018). *An investigation into factors affecting breeding success in the Tasmanian devil (Sarcophilus harrisii).* (Doctor of Philosophy). The University of Sydney,

SALE, M. G., & ARNOULD, J. P. Y. (2009). SPATIAL AND TEMPORAL ORGANIZATION IN THE SWAMP ANTECHINUS: COMPARISON BETWEEN ISLAND AND MAINLAND POPULATIONS. *Journal of Mammalogy, 90*(2), 347-355.

Sale, M. G., Kraaijeveld-Smit, F. J. L., & Arnould, J. P. Y. (2009). Natal dispersal and social organization of the swamp antechinus (Antechinus minimus) in a high-density island population. *Canadian Journal of Zoology, 87*(3), 262-272. doi:10.1139/z09-012

Sale, M. G., Kraaijeveld-Smit, F. J. L., & Arnould, J. P. Y. (2013). Multiple paternity in the swamp antechinus (Antechinus minimus). *Australian Mammalogy, 35*(2). doi:10.1071/am12039

Sale, M. G., Wilson, B. A., & Arnould, J. P. Y. (2008). Factors influencing population dynamics in island and mainland populations of the swamp antechinus (Antechinus minimus). *Australian Journal of Zoology, 56*, 187-194. doi:10.1071/ZO08076

Serena, M., & Soderquist, T. R. (1988). Growth and Development of Pouch Young of Wild and Captive *Dasyurus geoffroii* (Marsupialia : Dasyuridae). *Australian Journal of Zoology, 36*, 533-543. doi:<https://doi.org/10.1071/ZO9880533>

SERENA, M., & SODERQUIST, T. R. (1989). Spatial organization of a riparian population of the carnivorous marsupial Dasyurus geoffroii. *Journal of Zoology London, 2019*, 373-383. doi:doi.org/10.1111/j.1469-7998.1989.tb02586.x

Shimmin, G. A., Taggart, D. A., & Temple‐Smith, P. D. (2002). Mating behaviour in the agile antechinus *Antechinus agilis* (Marsupialia: Dasyuridae). *Journal of Zoology, 258*(1), 39-48. doi:10.1017/s0952836902001188

Soderquist, T. R. (1995). Ontogeny of Sexual Dimorphism in Size among Polytocous Mammals: Tests of Two Carnivorous Marsupials. *Journal of Mammalogy, 76*(2), 376-390. doi:<https://www.jstor.org/stable/1382349>

Soderquist, T. R., & Ealey, L. (1994). Social Interactions and Mating Strategies of a Solitary Carnivorous Marsupial, Phascogale tapoatafa, in the Wild. *Wildlife Research, 21*(5), 527-542. doi:doi.org/10.1071/WR9940527

Ward, S. J. (1990). Life History of the Feathertail Glider, Acrobates pygmaeus (Acrobatidae : Marsupialia) in South-eastern Australia. *Australian Journal of Zoology, 38*, 503-517.

Wooller, R. D., Richardson, K. C., Garavanta, C. A. M., Saffer, V. M., & Bryant, K. A. (2000). Opportunistic breeding in the polyandrous honey possum, Tarsipes rostratus. *Australian Journal of Zoology, 48*, 669-680. doi:10.1071/ZO00071

Woolley, P. A. (1991). Reproduction in Dasykaluta rosamondae (Marsupialia : Dasyuridae): Field and Laboratory Observations. *Australian Journal of Zoology, 39*, 549-568. doi:<https://doi.org/10.1071/ZO9910549>
