# Supplementary material for: Factors Affecting Multiple Paternity: Insights From the Eastern Quoll (Dasyurus viverrinus)
Source: Ecol Evol. 2026 Apr 30;16(5):e73515. doi: 10.1002/ece3.73515 (PMC13130336; doi:10.1002/ece3.73515)
Supplement: Supplementary file 1 — Figure S1: Phylogenetic relatedness and breeding system details for Australian marsupials. Phylogeny adapted from Westerman et al. (2016) and Krajewski et al. (2000). Values taken from sums using values presented by Dobson et al. (2018), are indicated by ^. Litter size provides the minimum and maximum number of possible offspring for a single litter, with the average number of offspring provided in (ellipses). Note two entries are present for Dasyurus hallucatus due to variation in different populations. Litters sired multiply indicated the percentage (%) of litters that have multiple fathers. Sires shows how many fathers have been observed in a single litter, with averages indicated in ellipses where available. Miscellaneous notes on parentage describes other aspects of maternity and paternity discussed by the relevant paper—notably, this includes paternity distribution within a litter. Female: Male weight is the percentage a female's weight is compared with a male (i.e., when males are larger than females, the value will be below 100%. A female of 10 g and a male of 20 g would result in 50%). Life Span describes the number of years a species lives in the wild. Aggression is a qualitative trait determined from descriptions in the literature: Low indicates that physical aggression was minimal, moderate that biting and some forced copulation occurs, and high indicates that biting is a regular feature of the species' breeding system, and may result in serious wounds. (M) indicates a male‐only trait, (F) a female‐only trait. Traits specific to the breeding season only are noted as Br, while traits that are specific to the non‐breeding season are indicated by NB. Where this study is reporting new information, it is written in bold. A* Indicates reproductive senescence, though individuals may survive beyond this point. Figure S2: Trends in the percentage of adult eastern quolls ( Dasyurus viverrinus ) breeding successfully in the Mulligans Flat Woodland Sanctuary populat [file ECE3-16-e73515-s001.zip › Supplementary file.docx]

# Supplementary information

# Supplementary Table 1

Supplementary Table 1. Model selection table for a binomial generalised linear mixed-model of eastern quoll (*Dasyurus viverrinus*) breeding success. The global model included weight (linear and quadratic terms), age, individual heterozygosity, sex, morph, and interactions between weight terms and age, with random effects of individual identity (id) and year. Models are ranked by AICc, with only models ΔAICc < 5 shown. A "+" indicates the variable was included in the model. Continuous variables (weight, age, heterozygosity) were scaled (mean-centered and standardised). The top-ranked model (ΔAICc = 0, shown in **bold**) was selected for inference. All models converged successfully. Model selection was conducted using the dredge function in MuMIn package (Barton, 2023). Random effects (individual id and year) were retained in all models.

| Global model call: glmmTMB(formula = breedingsuccess ~ (scale(weight) + I((scale(weight))^2)) * scale(age) + scale(indhet) + sex + morph + (1 \| id) + (1 \| year), data = pruned, family = "binomial", na.action = na.fail, ziformula = ~0, dispformula = ~1) | | | | | | | | | | | | | | | | | | | | |  |  |
| --- | --- | --- | --- | --- | --- | --- | --- | --- | --- | --- | --- | --- | --- | --- | --- | --- | --- | --- | --- | --- | --- | --- |
| cnd((Int)) | dsp((Int)) | cnd(I((scl(  wgh))^2)) | cnd(mrp) | | cnd(scl(age)) | | cnd(scl(ind)) | | cnd(scl(wgh)) | | cnd(sex) | | cnd(I((scl(wgh))^2):  scl(age)) | | df | | logLik | | AICc | | | delta |
| **1.187** | **+** | **-0.6925** | **+** | | **-1.19** | |  | | **2.505** | |  | |  | | **7** | | **-28.98** | | **73.7** | | | **0** |
| 1.434 | + | -0.8642 | + | | -1.584 | |  | | 2.325 | |  | | 0.6387 | | 9 | | -27.072 | | 75 | | | 1.32 |
| 1.286 | + | -0.9055 | + | | -1.421 | |  | | 2.508 | |  | | 0.1846 | | 8 | | -28.445 | | 75.1 | | | 1.46 |
| 1.111 | + | -0.6679 | + | | -1.253 | | -0.2827 | | 2.61 | |  | |  | | 8 | | -28.726 | | 75.7 | | | 2.02 |
| 1.222 | + | -0.6947 | + | | -1.192 | |  | | 2.534 | | + | |  | | 8 | | -28.975 | | 76.2 | | | 2.52 |
| 1.185 | + | -0.6748 | + | | -1.176 | |  | | 2.498 | |  | |  | | 8 | | -28.977 | | 76.2 | | | 2.52 |
| 0.7276 | + |  | + | | -1.067 | |  | | 2.096 | |  | |  | | 7 | | -30.466 | | 76.7 | | | 2.97 |
| 1.207 | + | -0.917 | + | | -1.563 | | -0.3968 | | 2.653 | |  | | 0.2242 | | 9 | | -27.995 | | 76.8 | | | 3.16 |
| 0.4103 | + |  | + | | -1.22 | |  | | 1.854 | |  | |  | | 6 | | -31.855 | | 77 | | | 3.3 |
| 1.289 | + | -0.8561 | + | | -1.584 | |  | | 2.187 | | + | | 0.6703 | | 10 | | -26.983 | | 77.5 | | | 3.83 |
| 1.299 | + | -0.9062 | + | | -1.421 | |  | | 2.519 | | + | | 0.1842 | | 9 | | -28.444 | | 77.7 | | | 4.06 |
| 1.183 | + | -0.6708 | + | | -1.263 | | -0.2969 | | 2.674 | | + | |  | | 9 | | -28.704 | | 78.3 | | | 4.58 |
| 1.112 | + | -0.674 | + | | -1.259 | | -0.2844 | | 2.613 | |  | |  | | 9 | | -28.726 | | 78.3 | | | 4.63 |
| 0.6801 | + |  | + | | -1.125 | | -0.2682 | | 2.228 | |  | |  | | 8 | | -30.206 | | 78.7 | | | 4.98 |
| Random terms (all models): cond(1 \| id), cond(1 \| year) | | | |  | |  | |  | |  | |  | |  | |  | |  | |  |  |  |

# Supplementary Table 2

Supplementary Table 2 Model selection table for binomial generalised linear mixed-model of female (top) and male (bottom) eastern quoll (*Dasyurus viverrinus*) breeding success. The global model included weight, age, individual heterozygosity, morph, with random effects of individual identity (id) and year. Models are ranked by AICc, with only models with ΔAICc < 5 shown. A "+" indicates the variable was included in the model. Continuous variables (weight, age, heterozygosity) were scaled (mean-centered and standardised). The top-ranked model (ΔAICc = 0) was selected for inference for males, while the top three models were selected for averaging and subsequent inference for females. All models converged successfully. Model selection was conducted using the dredge function in MuMIn package (Barton, 2023). Random effects (individual id and year) were retained in all models.

| **Female Breeding Success** | Global model call: glmmTMB(formula = breedingsuccess ~ scale(weight) + scale(age) + scale(indhet) + morph + (1 \| id) + (1 \| year), data = females, family = "binomial", na.action = na.fail, ziformula = ~0, dispformula = ~1) | | | | | | | | | | | | | | | | | | | | | | | | |
| --- | --- | --- | --- | --- | --- | --- | --- | --- | --- | --- | --- | --- | --- | --- | --- | --- | --- | --- | --- | --- | --- | --- | --- | --- | --- |
|  | cnd((Int)) | dsp((Int)) | | cnd(mrp) | | cnd(scl(age)) | | cnd(scl(ind)) | | | | cnd(scl(wgh)) | | | | | df | | logLik | | AICc | | delta | | weight |
|  | **-0.5225** | **+** | | **+** | | **-0.9865** | |  | | | | **1.484** | | | | | **6** | | **-17.638** | | **49.5** | | **0** | | **0.3** |
|  | **-1.199** | **+** | |  | | **-0.9376** | |  | | | | **1.383** | | | | | **5** | | **-19.283** | | **50.1** | | **0.6** | | **0.222** |
|  | **-1.202** | **+** | |  | | **-1.045** | | **-0.4641** | | | | **1.473** | | | | | **6** | | **-18.59** | | **51.5** | | **1.9** | | **0.116** |
|  | -0.6349 | + | | + | |  | |  | | | | 1.377 | | | | | 5 | | -20.067 | | 51.7 | | 2.17 | | 0.102 |
|  | -0.5906 | + | | + | | -1.082 | | -0.34 | | | | 1.508 | | | | | 7 | | -17.337 | | 51.8 | | 2.24 | | 0.098 |
|  | -1.13 | + | |  | |  | |  | | | | 1.27 | | | | | 4 | | -21.402 | | 51.8 | | 2.28 | | 0.096 |
|  | -1.136 | + | |  | |  | | -0.294 | | | | 1.302 | | | | | 5 | | -21.053 | | 53.7 | | 4.14 | | 0.038 |
|  | -0.669 | + | | + | |  | | -0.1379 | | | | 1.375 | | | | | 6 | | -20.001 | | 54.3 | | 4.72 | | 0.028 |
|  | Random terms (all models): cond(1 \| id), cond(1 \| year) | | | | | | | | | | |  | | | | |  | |  | |  | |  | |  |
| **Male breeding success** | Global model call: glmmTMB(formula = breedingsuccess ~ scale(weight) + scale(age) + morph + (1 \| id) + (1 \| year), data = males, family = "binomial", na.action = na.fail, ziformula = ~0, dispformula = ~1) | | | | | | | | | | | | | | | | | | | | | | | | |
|  | cnd((Int)) | | dsp((Int)) | | cnd(mrp) | | cnd(scl(age)) | | | cnd(scl(wgh)) | | | | df | | logLik | | | | AICc | | delta | | weight | |
|  | **1.952** | | **+** | | **+** | | **-1.544** | | | **1.967** | | | | **6** | | **-12.283** | | | | **40.4** | | **0** | | **0.52** | |
|  | 1.301 | | + | | + | |  | | | 1.028 | | | | 5 | | -15.297 | | | | 43.2 | | 2.82 | | 0.127 | |
|  | 0.7917 | | + | |  | | -1.458 | | | 1.976 | | | | 5 | | -15.342 | | | | 43.3 | | 2.91 | | 0.122 | |
|  | 1.508 | | + | | + | |  | | |  | | | | 4 | | -17.03 | | | | 43.7 | | 3.34 | | 0.098 | |
|  | 0.5738 | | + | |  | |  | | | 0.8963 | | | | 4 | | -17.114 | | | | 43.9 | | 3.51 | | 0.09 | |
|  | 0.5202 | | + | |  | |  | | |  | | | | 3 | | -19.203 | | | | 45.4 | | 4.98 | | 0.043 | |
|  | Random terms (all models): cond(1 \| id), cond(1 \| year) | | | | | | | |  | |  | |  | |  | | |  | | | | | | | |

# Supplementary Table 3

Supplementary Table 3. Model selection table for poisson-based generalised linear mixed-model of eastern quoll (*Dasyurus viverrinus*) fecundity. The global model included weight, age, individual heterozygosity, morph, with random effects of individual identity (id) and year. Models are ranked by AICc, with only models with ΔAICc < 5 shown. A "+" indicates the variable was included in the model. Continuous variables (weight, age, heterozygosity) were scaled (mean-centered and standardised). All models converged successfully. Model selection was conducted using the dredge function in MuMIn package (Barton, 2023). Random effects (individual id and year) were retained in all models.

| Global model call: glmmTMB (formula = offspring ~ I((scale(weight))^2) * scale(age) + scale(weight) + scale(age) + scale(indhet) + morph + sex + (1 \| id) + (1 \| year), data = success, family = "poisson", na.action = na.fail, ziformula = ~0, dispformula = ~1) | | | | | | | | | | | | |
| --- | --- | --- | --- | --- | --- | --- | --- | --- | --- | --- | --- | --- |
| cnd((Int)) | dsp((Int)) | cnd(I((scl(wgh))^2)) | cnd(mrp) | cnd(scl(age)) | cnd(scl(ind)) | cnd(scl(wgh)) | cnd(sex) | df | logLik | AICc | delta | weight |
| **0.972** | **+** |  |  |  |  |  |  | **3** | **-61.351** | **129.6** | **0** | **0.215** |
| 0.773 | + |  |  |  |  |  | + | 4 | -60.54 | 130.6 | 1.03 | 0.128 |
| 0.985 | + |  |  |  |  | 0.144 |  | 4 | -60.905 | 131.3 | 1.76 | 0.089 |
| 0.9817 | + |  |  | -0.105 |  |  |  | 4 | -61.218 | 132 | 2.38 | 0.065 |
| 0.9757 | + |  |  |  | 0.07 |  |  | 4 | -61.222 | 132 | 2.39 | 0.065 |
| 1.025 | + | -0.051 |  |  |  |  |  | 4 | -61.288 | 132.1 | 2.52 | 0.061 |
| 0.9707 | + |  | + |  |  |  |  | 4 | -61.351 | 132.2 | 2.65 | 0.057 |
| 1.177 | + | -0.204 |  |  |  | 0.307 |  | 5 | -60.064 | 132.5 | 2.94 | 0.049 |
| 0.9818 | + |  |  | -0.221 |  | 0.257 |  | 5 | -60.12 | 132.6 | 3.05 | 0.047 |
| 0.7842 | + |  |  | -0.105 |  |  | + | 5 | -60.397 | 133.2 | 3.6 | 0.035 |
| 0.8249 | + | -0.057 |  |  |  |  | + | 5 | -60.44 | 133.3 | 3.69 | 0.034 |
| 0.7811 | + |  |  |  | 0.021 |  | + | 5 | -60.527 | 133.5 | 3.86 | 0.031 |
| 0.7849 | + |  |  |  |  | 0.015 | + | 5 | -60.537 | 133.5 | 3.88 | 0.031 |
| 0.7749 | + | + | + |  |  |  | + | 5 | -60.539 | 133.5 | 3.89 | 0.031 |
| 0.9854 | + |  |  |  | 0.034 | 0.132 |  | 5 | -60.878 | 134.2 | 4.56 | 0.022 |
| 0.9821 | + | + | + |  |  | 0.0144 |  | 5 | -60.904 | 134.2 | 4.62 | 0.021 |
| 0.9855 | + |  |  | -0.11 | 0.075 |  |  | 5 | -61.072 | 134.5 | 4.95 | 0.018 |
| Random terms (all models): cond(1 \| id), cond(1 \| year) | | | | | | | | | | | | |

# Supplementary Table 4

Supplementary Table 4. Eastern quoll (*Dasyurus viverrinus*) pedigree for a population within Mulligans Flat Woodland Sanctuary (MFWS), ACT, Australia. Pedigree construction occurred in Colony v 2.0.6.6, and was informed by 1745 single nucleotide polymorphisms and demographic data obtained through long term monitoring of the MFWS population. Birth year for each “Offspring” born within MFWS was assigned based on age at first capture, and impossible parents (i.e. individuals not yet born based on age at first capture, individuals not yet translocated into the MFWS population, or individuals that would have dies due to old age) excluded from consideration. Where data was available, offspring were assigned to their known mother. The pedigree-assigned “Mother” and “Father” for each MFWS individual were then recorded, and for each year (i.e. 2016, 2017, etc) all offspring of a single female were grouped into “Litters”.

| **Litter** | **Offspring** | **Born** | **Father** | **Mother** | **Litter** | **Offspring** | **Born** | **Father** | **Mother** | **Litter** | **Offspring** | **Born** | **Father** | **Mother** |
| --- | --- | --- | --- | --- | --- | --- | --- | --- | --- | --- | --- | --- | --- | --- |
| **1** | 0DD31 | 2016 | 3 | 1 | **14** | FD4E9 | 2017 | 1 | 180F7 | **28** | 83C33 | 2018 | 7 | 83CAB |
| **2** | 83B4C | 2016 | 3 | 2 | **14** | BBC6C | 2017 | FC42B | 180F7 | **28** | 88D4D | 2018 | 7 | 83CAB |
| **3** | 84E6F | 2016 | 10 | 3 | **15** | 0DC56 | 2017 | F1041 | 1BEFa | **29** | 5156 | 2018 | 0DD54 | BDD65 |
| **4** | 0E33F | 2016 | 18244 | 88C2Ba | **15** | FD8C7 | 2017 | F1041 | 1BEFa | **29** | 840B6 | 2018 | 0DD54 | BDD65 |
| **4** | C1291 | 2016 | 18244 | 88C2Ba | **16** | 00D36 | 2017 | F1041 | 84E6F | **30** | 84223 | 2018 | 1 | D849 |
| **5** | F1041 | 2016 | 2 | E8FC0 | **16** | 0E0A7 | 2017 | F1041 | 84E6F | **30** | 3B6DE | 2018 | BBC6C | D849 |
| **5** | F1b | 2016 | 2 | E8FC0 | **17** | 88C7E | 2017 | 3 | BF9FC | **31** | 94FF7 | 2018 | 0DC56 | FD3E9 |
| **5** | F1a | 2016 | 18244 | E8FC0 | **17** | 8BD0A | 2017 | FC42B | BF9FC | **31** | AA9A3 | 2018 | 0DC56 | FD3E9 |
| **5** | FC07B | 2016 | 18244 | E8FC0 | **17** | BDD65 | 2017 | FC42B | BF9FC | **31** | 94D44 | 2018 | F5B5E | FD3E9 |
| **5** | 886C3 | 2016 | E91DB | E8FC0 | **17** | FD5C1 | 2017 | FC42B | BF9FC | **32** | 94AEC | 2018 | F5B5E | FD54D |
| **6** | 88761 | 2016 | 18244 | FF0C7 | **18** | 00B49 | 2017 | 4 | C682 | **32** | 94A21 | 2018 | FD4E9 | FD54D |
| **6** | FC42B | 2016 | 18244 | FF0C7 | **19** | 007AE | 2017 | FC42B | FD6C1 | **32** | 94B62 | 2018 | FD4E9 | FD54D |
| **6** | FD912 | 2016 | 18244 | FF0C7 | **19** | CD453 | 2017 | FC42B | FD6C1 | **33** | 39DE4 | 2019 | 3B6DE | 84143 |
| **6** | F36F0 | 2016 | E91DB | FF0C7 | **20** | FD3E9 | 2017 | 18244 | FD912 | **33** | AA6C4 | 2019 | 94AEC | 84143 |
| **7** | 83AFE | 2017 | F1041 | 4 | **20** | FD54D | 2017 | F1041 | FD912 | **33** | D0E214 | 2019 | C062B | 84143 |
| **8** | 0DD92 | 2017 | F1041 | 3051 | **21** | 83C84 | 2018 | 6 | 5 | **33** | 7C787 | 2019 | F5B5E | 84125 |
| **8** | 0DF4C | 2017 | F1041 | 3051 | **21** | AA8AF | 2018 | 6 | 5 | **34** | AA8B8 | 2019 | 0DF4C | 7C5C5 |
| **8** | FDC0D | 2017 | F1041 | 3051 | **21** | AAB3F | 2018 | 6 | 5 | **34** | 7AE21 | 2019 | C062B | 7C5C5 |
| **9** | F5B5E | 2017 | F1041 | 5340 | **21** | AD378 | 2018 | 6 | 5 | **35** | 94C36 | 2019 | F1041 | 94FF7 |
| **9** | FDA82 | 2017 | F1041 | 5340 | **22** | 7B8A2 | 2018 | BBC6C | 6 | **36** | 00B5F | 2019 | C062B | AD30A |
| **10** | 0E2A2 | 2017 | F1041 | 88761 | **23** | 83EAC | 2018 | 0DD54 | 1715 | **36** | 7B551 | 2019 | F5B5E | AD30A |
| **10** | C062B | 2017 | F1041 | 88761 | **23** | AD46F | 2018 | 0DD54 | 1715 | **36** | 94BD9 | 2019 | F5B5E | AD30A |
| **10** | 0DD4C | 2017 | FC42B | 88761 | **23** | AD30A | 2018 | 0DF4C | 1715 | **37** | 7BAD9 | 2019 | 88C7E | FD3E9 |
| **11** | BDDFC | 2017 | 88C7E | 0DD31 | **24** | 951ED | 2018 | 0DC56 | 0DD31 | **37** | 7C63B | 2019 | BBC6C | FD3E9 |
| **12** | 0DD54 | 2017 | 5 | 0DDE1 | **24** | AD42A | 2018 | BBC6C | 0DD31 | **37** | 7B630 | 2019 | C062B | FD3E9 |
| **12** | 950C9 | 2017 | 5 | 0DDE1 | **25** | 840FA | 2018 | 0DF4C | 0DD4C | **38** | 738 | 2019 | 11 | FD536 |
| **12** | FD705 | 2017 | 5 | 0DDE1 | **25** | FF6FE | 2018 | 0DF4C | 0DD4C | **38** | 7C60E | 2019 | 11 | FD536 |
| **12** | FD716 | 2017 | 5 | 0DDE1 | **25** | FECC6 | 2018 | 88C7E | 0DD4C | **39** | 7B1E4 | 2019 | 0DF4C | FD895 |
| **12** | 3B9C5 | 2017 | 8 | 0DDE1 | **26** | FD895 | 2018 | 0DC56 | 0DD92 | **39** | 7B774 | 2019 | F1041 | FD895 |
| **12** | 0DE28 | 2017 | 9 | 0DDE1 | **26** | 7C5C5 | 2018 | C062B | 0DD92 | **40** | FDB15 | 2020 | 94AEC | 7B8A2 |
| **13** | 00CC5 | 2017 | F1041 | 0E33F | **26** | 83DBF | 2018 | C062B | 0DD92 | **40** | FDB53 | 2020 | BBC6C | 180F7 |
| **13** | FDBDD | 2017 | F1041 | 0E33F | **26** | 84125 | 2018 | FDC0D | 0DD92 | **41** | 83B1A | 2020 | AA6C4 | 94C36 |
| **13** | D0E314 | 2017 | FC42B | 0E33F | **27** | 84143 | 2018 | 0DC56 | 83AFE | **41** | BD661 | 2020 | AA6C4 | 94C36 |
|  |  |  |  |  |  |  |  |  |  | **42** | FD7B0 | 2020 | 00B5F | AD30A |

# Supplementary Table 5

Supplementary Table 5 Comparison of litter size and multiple paternity within the eastern quoll (*Dasyurus viverrinus*) population at Mulligans Flat Woodland Sanctuary (MFWS), ACT, Australia. Data derived from a pedigree constructed in Colony v 2.0.6.6. Pedigree construction was informed by 1745 single nucleotide polymorphisms and demographic data obtained through long term monitoring of the MFWS population. “Litter size” represents various subsets of the total dataset based on the minimum number of offspring in a litter, and “Count” displays the number of litters that were reconstructed in the pedigree. The frequency of multiple paternity is represented by the “% litters with multiple paternity”, being a count of how many litters within the subset had multiple fathers assigned in the pedigree. The “Mean number of sires” presents the average number of fathers assigned to litters within the pedigree.

| ***Litter size and multiple paternity in the eastern quoll (Dasyurus viverrinus)*** | | | | |
| --- | --- | --- | --- | --- |
| **Litter Size** | ***Count*** | ***Count of litters with***  ***multiple paternity*** | ***% litters with***  ***multiple paternity*** | ***Mean number of sires*** |
| ***1 -6 young*** | 42 | 20 | *47.62%* | 1.62 |
| ***2-6 young*** | 32 | 20 | 62.50% | 1.81 |
| ***3-6 young*** | 16 | 14 | 87.50% | 2.25 |
| ***4-6 young*** | 7 | 6 | 85.71% | 2.57 |
| ***5-6 young*** | 2 | 2 | 100% | 3 |
| ***6 young*** | 1 | 1 | 100% | 3 |

# Supplementary Table 6

Supplementary Table 6. Reproductive patterns in a population of reintroduced eastern quolls (*Dasyurus viverrinus*) at Mulligans Flat Woodland Sanctuary (MFWS), ACT, Australia. Parentage data obtained from a pedigree constructed in Colony v 2.0.6.6, informed by 1745 single nucleotide polymorphisms and demographic data obtained through long term monitoring of the MFWS population. R^2^ and p values obtained through simple linear regression.

| **Multiple paternity across time in Mulligans Flat Woodland Sanctuary** | | | | | | | | | | |
| --- | --- | --- | --- | --- | --- | --- | --- | --- | --- | --- |
| **Year** | **Cohort** | **Count Offspring recruited in MFWS** | **Median litter size** | **Mean litter size** | **Median sires/litter** | **Mean sires/litter** | **Litters with**  **multiple paternity** | **For >1 young,**  **litters with**  **multiple paternity** | ***Effect of year on multiple paternity frequency (>1 young)*** | |
| **2016** | **MFWS 1** | 11 | 3 | 2.75 | 1.5 | 1.5 | 33.3% | 66.7% | R^2^ | 0.51 |
| **2017** | **MFWS 2** | 37 | 2.5 | 2.64 | 1 | 1.29 | 42.9% | 54.5% | p | 0.286 |
| **2018** | **MFWS 3** | 30 | 2 | 2.5 | 1.5 | 1.58 | 58.3% | 70% | ***Effect of year on mean number of sires*** | |
| **2019** | **MFWS 4** | 18 | 2 | 1.8 | 1 | 1.5 | 71.4% | 83.3% | R^2^ | 0.09 |
| **2020** | **MFWS 5** | 4 | 1 | 1.33 | 1 | 1 | 0 | n/a | p | 0.699 |

| **Parentage patterns across time** | | | | | | | | | | | |
| --- | --- | --- | --- | --- | --- | --- | --- | --- | --- | --- | --- |
| **Year** | **Cohort** | **Extant adult females** | **Count of mothers** | **Females = Mothers** | **Median offspring/mother** | **Mean**  **offspring/mother** | **Extant adult**  **males** | **Count of**  **fathers** | **Males = Fathers** | **Median offspring/father** | **Mean offspring/Father** |
| **2016** | **MFWS 1** | 8 | 4 | 50% | 3 | 2.75 | 3 | 3 | 100% | 2 | 3.67 |
| **2017** | **MFWS 2** | 31 | 14 | 45.16% | 2.5 | 2.64 | 14 | 9 | 64.29% | 1 | 4.11 |
| **2018** | **MFWS 3** | 51 | 12 | 23.53% | 2 | 2.5 | 35 | 11 | 31.43% | 2 | 2.73 |
| **2019** | **MFWS 4** | 53 | 10 | 18.87% | 2 | 1.8 | 31 | 11 | 35.48% | 1 | 1.64 |
| **2020** | **MFWS 5** | 39 | 3 | 7.69% | 1 | 1.33 | 10 | 3 | 30.00% | 1 | 1.33 |

# Supplementary Table 7

Supplementary Table 7. Model diagnostic tables for investigation into eastern quoll (*Dasyurus viverrinus*) breeding systems. Results of the check_collinearity and check_overdispersion functions of the *performance* package v0.15.3), in R v4.5.0.

| **Binomial model of breeding success - collinearity** | | | | | | | | | | | | | | |  |  |  |  |  |
| --- | --- | --- | --- | --- | --- | --- | --- | --- | --- | --- | --- | --- | --- | --- | --- | --- | --- | --- | --- |
| **Term** | **VIF** | **VIF_CI_low** | | | **VIF_CI_high** | | **SE_factor** | | **Tolerance** | | **Tolerance_CI_low** | | **Tolerance_CI_high** | | **Overdispersion** | | | |  |
| I((scale(weight))^2) | 2.580 | 1.918 | | | 3.720 | | 1.606 | | 0.388 | | 0.269 | | 0.521 | | **Metric** | | **Value** | |  |
| morph | 1.457 | 1.193 | | | 2.085 | | 1.207 | | 0.686 | | 0.480 | | 0.838 | | Dispersion ratio | | 0.980 | |  |
| scale(age) | 1.523 | 1.233 | | | 2.172 | | 1.234 | | 0.657 | | 0.460 | | 0.811 | | p-value | | 0.936 | |  |
| scale(weight) | 3.063 | 2.235 | | | 4.445 | | 1.750 | | 0.326 | | 0.225 | | 0.447 | |  |  |  |  |  |
| **Poisson model of individual fecundity** | | | | | | | | | | | | | | | |  |  |  |  |
| **Term** | | | **VIF** | **VIF_CI_low** | | **VIF_CI_high** | | **SE_factor** | | **Tolerance** | | **Tolerance_CI_low** | | **Tolerance_CI_high** | | **Overdispersion** | | | |
| I((scale(weight))^2) | | | 1.547 | 1.220 | | 2.365 | | 1.244 | | 0.646 | | 0.423 | | 0.820 | | **Metric** | | **Value** | |
| scale(age) | | | 1.918 | 1.439 | | 2.919 | | 1.385 | | 0.521 | | 0.343 | | 0.695 | | Dispersion ratio | | 0.529 | |
| scale(weight) | | | 1.933 | 1.448 | | 2.942 | | 1.390 | | 0.517 | | 0.340 | | 0.690 | | Pearson's Chi-Squared | | 11.115 | |
| scale(indhet) | | | 1.188 | 1.033 | | 2.079 | | 1.090 | | 0.842 | | 0.481 | | 0.968 | | p-value | | 0.960 | |
| morph | | | 1.064 | 1.001 | | 5.231 | | 1.032 | | 0.940 | | 0.191 | | 0.999 | |  |  |  |  |
| sex | | | 1.657 | 1.284 | | 2.523 | | 1.287 | | 0.603 | | 0.396 | | 0.779 | |  |  |  |  |
| I((scale(weight))^2):scale(age) | | | 2.214 | 1.619 | | 3.381 | | 1.488 | | 0.452 | | 0.296 | | 0.618 | |  |  |  |  |

| **Binomial model of breeding success – by sex** | | | | | | | | |  |  |
| --- | --- | --- | --- | --- | --- | --- | --- | --- | --- | --- |
| **female** | **Term** | **VIF** | **VIF_CI_low** | **VIF_CI_high** | **SE_factor** | **Tolerance** | **Tolerance_CI_low** | **Tolerance_CI_high** | **Overdispersion** | |
|  | scale(weight) | 1.121 | 1.009 | 2.532 | 1.059 | 0.892 | 0.395 | 0.991 | Dispersion ratio | 0.988 |
|  | scale(age) | 1.173 | 1.025 | 2.181 | 1.083 | 0.852 | 0.458 | 0.975 |  |  |
|  | scale(indhet) | 1.154 | 1.019 | 2.253 | 1.074 | 0.866 | 0.444 | 0.981 | p-value | 0.984 |
|  | morph | 1.093 | 1.004 | 3.158 | 1.046 | 0.915 | 0.317 | 0.996 |  |  |
| Males | **Term** | **VIF** | **VIF_CI_low** | **VIF_CI_high** | **SE_factor** | **Tolerance** | **Tolerance_CI_low** | **Tolerance_CI_high** | **Overdispersion** | |
|  | morph | 2.547 | 1.724 | 4.306 | 1.596 | 0.393 | 0.232 | 0.580 | Dispersion ratio | 0.906 |
|  | scale(age) | 3.889 | 2.474 | 6.664 | 1.972 | 0.257 | 0.150 | 0.404 |  |  |
|  | scale(weight) | 3.286 | 2.136 | 5.601 | 1.813 | 0.304 | 0.179 | 0.468 | p-value | 0.792 |

# Supplementary Table 8

Supplementary Table 8. Reciprocal cross data from eastern quolls (Dasyurus viverrinus) born into the Mulligans Flat Woodland Sanctuary, Canberra ACT, between 2017 and 2020. Data derived using 1745 single-nucleotide polymorphisms and long-term demographic data to reconstruct a pedigree in Colony v 2.0.6.6

| Parental cross | | Count | | | % Offspring Black | % Offspring Fawn |
| --- | --- | --- | --- | --- | --- | --- |
| Father | Mother | Incidences | Black Offspring | Fawn Offspring |  |  |
| Black | Black | 30 | 27 | 3 | 90 | 10 |
| Fawn | Black | 20 | 9 | 11 | 45 | 55 |
| Black | Fawn | 13 | 4 | 9 | 30.8 | 69.2 |
| Fawn | Fawn | 5 | 2 | 3 | 40 | 60 |
